# A novel sORF gene mutant strain of *Yersinia pestis* vaccine EV76 offers enhanced safety and improved protection against plague

**DOI:** 10.1371/journal.ppat.1012129

**Published:** 2024-03-28

**Authors:** Xiao Guo, Youquan Xin, Zehui Tong, Shiyang Cao, Yuan Zhang, Gengshan Wu, Hongyan Chen, Tong Wang, Yajun Song, Qingwen Zhang, Ruifu Yang, Zongmin Du

**Affiliations:** 1 School of Basic Medical Sciences, Anhui Medical University Hefei, China; 2 State Key Laboratory of Pathogen and Biosecurity, Beijing Institute of Microbiology and Epidemiology, Beijing, China; 3 Key Laboratory for Plague Prevention and Control of Qinghai Province, Qinghai Institute for Endemic Disease Prevention and Control, Xining, China; Children’s Hospital Boston, UNITED STATES

## Abstract

We recently identified two virulence-associated small open reading frames (sORF) of *Yersinia pestis*, named *yp1* and *yp2*, and null mutants of each individual genes were highly attenuated in virulence. Plague vaccine strain EV76 is known for strong reactogenicity, making it not suitable for use in humans. To improve the immune safety of EV76, three mutant strains of EV76, Δ*yp1*, Δ*yp2*, and Δ*yp1&yp2* were constructed and their virulence attenuation, immunogenicity, and protective efficacy in mice were evaluated. All mutant strains were attenuated by the subcutaneous (*s*.*c*.) route and exhibited more rapid clearance in tissues than the parental strain EV76. Under iron overload conditions, only the mice infected with EV76Δ*yp1* survived, accompanied by less draining lymph nodes damage than those infected by EV76. Analysis of cytokines secreted by splenocytes of immunized mice found that EV76Δ*yp2* induced higher secretion of multiple cytokines including TNF-α, IL-2, and IL-12p70 than EV76. On day 42, EV76Δ*yp2* or EV76Δ*yp1&yp2* immunized mice exhibited similar protective efficacy as EV76 when exposed to *Y*. *pestis* 201, both via *s*.*c*. or intranasal (*i*.*n*.) routes of administration. Moreover, when exposed to 200–400 LD_50_ *Y*. *pestis* strain 201Δ*caf1* (non-encapsulated *Y*. *pestis*), EV76Δ*yp2* or EV76Δ*yp1&yp2* are able to afford about 50% protection to *i*.*n*. challenges, significantly better than the protection afforded by EV76. On 120 day, mice immunized with EV76Δ*yp2* or EV76Δ*yp1&yp2* cleared the *i*.*n*. challenge of *Y*. *pestis* 201-*lux* as quickly as those immunized with EV76, demonstrating 90–100% protection. Our results demonstrated that deletion of the *yp2* gene is an effective strategy to attenuate virulence of *Y*. *pestis* EV76 while improving immunogenicity. Furthermore, EV76Δ*yp2* is a promising candidate for conferring protection against the pneumonic and bubonic forms of plague.

## Author summary

*Y*. *pestis*, the causative agent of the deadly plague, has caused three global pandemics throughout history and continues to pose a significant threat in various regions of the world. Unfortunately, there are currently no approved vaccines available to safeguard humans against *Y*. *pestis* infection. While the live-attenuated *Y*. *pestis* EV76 vaccine strain has been utilized in high-risk populations in China, Mongolia, and former Soviet countries, its usage is restricted due to potential severe adverse effects. In a recent study, we identified two small open reading frames (sORF)-encoded peptides of *Y*. *pestis*, namely SEP-yp1 and SEP-yp2, which are associated with the bacterium’s virulence. To improve the safety and efficacy of the EV76 vaccine, we constructed three mutant strains (Δ*yp1*, Δ*yp2*, and Δ*yp1&yp2*) and evaluated their virulence attenuation, immunogenicity, and ability to protect against *Y*. *pestis* infection in mice. Our findings demonstrated that deleting the *yp2* gene was a successful approach to diminish the virulence of *Y*. *pestis* EV76 while simultaneously enhancing its ability to provoke an immune response. Furthermore, our study revealed that EV76Δ*yp2* exhibited superior protection against different forms of plague, including pneumonic and bubonic, making it a promising candidate for further development as a plague vaccine.

## Introduction

Plague caused by *Yersinia pestis* is a fulminant infectious disease, resulting in nearly 200 million deaths in human history [1,2]. Plague presents itself in various clinical forms, namely bubonic, pneumonic, and septicemic plague. It is commonly transmitted among rodents through flea bites, usually leading to bubonic plague, which can progress to septicemic plague as the disease advances. Pneumonic plague is the most lethal form, and it can be transmitted among humans through the inhalation of infectious aerosols. The incubation period (1–3 days) of pneumonic plague is short, followed by a rapid disease progression that is often associated with high mortality [3]. Although plague endemics have been successfully controlled over the last half century, the 2017 plague epidemic in Madagascar was a reminder that *Y*. *pestis* remains a real threat in many parts of the world [4]. There are still several hundred human cases annually, scattered in different countries in Africa, the Americas, and Asia [5,6]. Unfortunately, antibiotic-resistant *Y*. *pestis* strains have already emerged in different countries in the meanwhile [7–10]. Due to its extreme lethality and ability to be transmitted via aerosol, *Y*. *pestis* is categorized as a Tier 1 select agent, or one of the few agents most likely to be utilized as a biological weapon [11]. Vaccination is the most effective and economical method to prevent plague, but there are currently no World Health Organization-recommended vaccines to protect human population against plague [12]. Thus, developing a safe and effective vaccine against plague remains of high interest.

Those currently under clinical trials are all subunit vaccines based on two antigens: F1 (capsular antigen) and LcrV (low calcium response V antigen, a type 3 secretion system [T3SS] component) [13–16]. Non-encapsulated (F1-negative) *Y*. *pestis* strains are widespread in nature, about 10–16% in a field sampling study [17], and this type of strain remains fully virulent [18–20]. Likewise, LcrV exists in at least five clades in the *Yersinia* species, and antibody responses to these LcrV variants are not cross-protective [21]. Therefore, there is always a concern that F1 and LcrV-based subunit vaccines may not provide complete protection against all *Y*. *pestis* strains [22].

Compared to other types of plague vaccines, live-attenuated plague vaccines possess a distinct advantage in rapidly activating both humoral and cellular immunity, [23,24], eliciting immunity against numerous antigens [25], and the immunoprotective effect can last for 10–12 months [26,27]. The live-attenuated *Y*. *pestis* EV76 vaccine strain has been used in populations at high-risk exposure in China, Mongolia, and former Soviet countries. EV76 is highly attenuated due to the absence of the pigmentation locus (*pgm*^*-*^*)* that is responsible for acquisition of host iron [28,29], and it can induce protection against both bubonic plague and pneumonic plague [30]. However, EV76 vaccine potentially causes major side effects, such as severe headaches, pyrexia, and general malaise [30]. It has been shown that *pgm*^-^ vaccine strains can restore virulence under conditions of iron overload [31,32]. There is also a case report of death in a patient with hereditary hemochromatosis caused by infection with an attenuated strain of *pgm*- *Y*. *pestis* [31,33,34]. Thus, EV76 vaccine has not been approved to be used worldwide due to its strong reactogenicity.

Our previous study has identified two small open reading frames (sORF)-encoded peptides (SEPs) of *Y*. *pestis*, namely SEP-yp1 and SEP-yp2. Deleting either of them in *Y*. *pestis* 201 strain results in a significant reduction in the intracellular survival capability and virulence in mice [35]. Specifically, *s*.*c*. challenge with approximate 100 CFU did not results in the death of mice challenged with the *yp2* mutant and only caused death of 25% of mice challenged with the *yp1* mutant. These findings suggest that *yp1* and *yp2* mutants exhibit significantly reduced virulence in mice. To determine whether the side effects of EV76 vaccine can be lowered while maintaining its immune protections by introducing these mutations, we construct mutants of EV76 by deleting one of SEP-yp1 and SEP-yp2 encoding genes, *yp1* or *yp2*, or both of them and evaluate the residual virulence of these mutant strains, including their safety under iron overload condition. We further compared different sORF genes mutant strains and EV76 vaccine in a short-term study and a long-term study for their protective efficacies. Our results show that the deletion of sORF genes further lowers the residual virulence of EV76. EV76Δ*yp2* induces a stronger cellular immune response than EV76 and EV76Δ*yp1&yp2*. Moreover, EV76Δ*yp2* and EV76Δ*yp1&yp2* afford better protection against *i*.*n*. challenge of non-encapsulated *Y*. *pestis* than EV76 after two doses of immunization.

## Results

### Construction of sORF genes deletion mutants of *Y*. *pestis*

To further reduce the residual virulence of EV76 and improve the vaccine safety, we deleted the *yp1* and (or) *yp2* genes from EV76, generating different mutants named EV76Δ*yp1*, EV76Δ*yp2*, and EV76Δ*yp1&yp2* (S1 Table). The in-frame deletion of the *yp1* and (or) *yp2* genes from EV76 was confirmed by PCR using specific primers (S2 Table) as well as by DNA sequencing of the PCR products flanking the *yp1* and *yp2* genes (S1A and S1B Fig). To determine the expression of the major protective antigen in these mutants of EV76, the expression of F1 and LcrV was detected by Western blot (S1C and S1D Fig). No obvious difference was found between the three mutants and EV76.

### Characterization of the residual virulence, immunogenicity, and safety of the sORF genes mutant strains of EV76

To further confirm the contributions of *yp1* and *yp2* to the pathogenicity of *Y*. *pestis*, we determined the median lethal dose (LD_50_) of 201 mutants with deletion of either *yp1*, *yp2* or both genes. Table 1 displays the LD_50_ values of these *Y*. *pestis* 201 mutants in BALB/c mice (6–8 weeks old, n = 5 or 10 per group) challenged subcutaneously. All the *Y*. *pestis* 201 mutants exhibited a significant attenuation in virulence, with as increase in LD_50_ more than 10^4^-folds, suggesting that *yp1* and *yp2* are critical for the virulence of *Y*. *pestis* (S2 Fig).

**Table 1:** Virulence attenuation in BALB/c mice of *Y*. *pestis* 201 sORF mutants.

Strain	Characteristics	LD_50_ by *s*.*c*. Route (CFU)^a^	References
201	wild type	3.1	[36]
201Δ*yp1*	in-frame deletion of *yp1*	4.8×10^4^	[36]
201Δ*yp2*	in-frame deletion of *yp2*	9.0×10^4^	This study^b^
201Δ*yp1&yp2*	in-frame deletion of *yp1* and *yp2*	5.2×10^4^	This study^b^

^a^ The confidence interval was unbounded.

^b^ Survival curves of mice *s*.*c* challenged with different doses of *Y*. *pestis* 201 sORF mutant strains were shown in S2 Fig.

**Fig 1:**
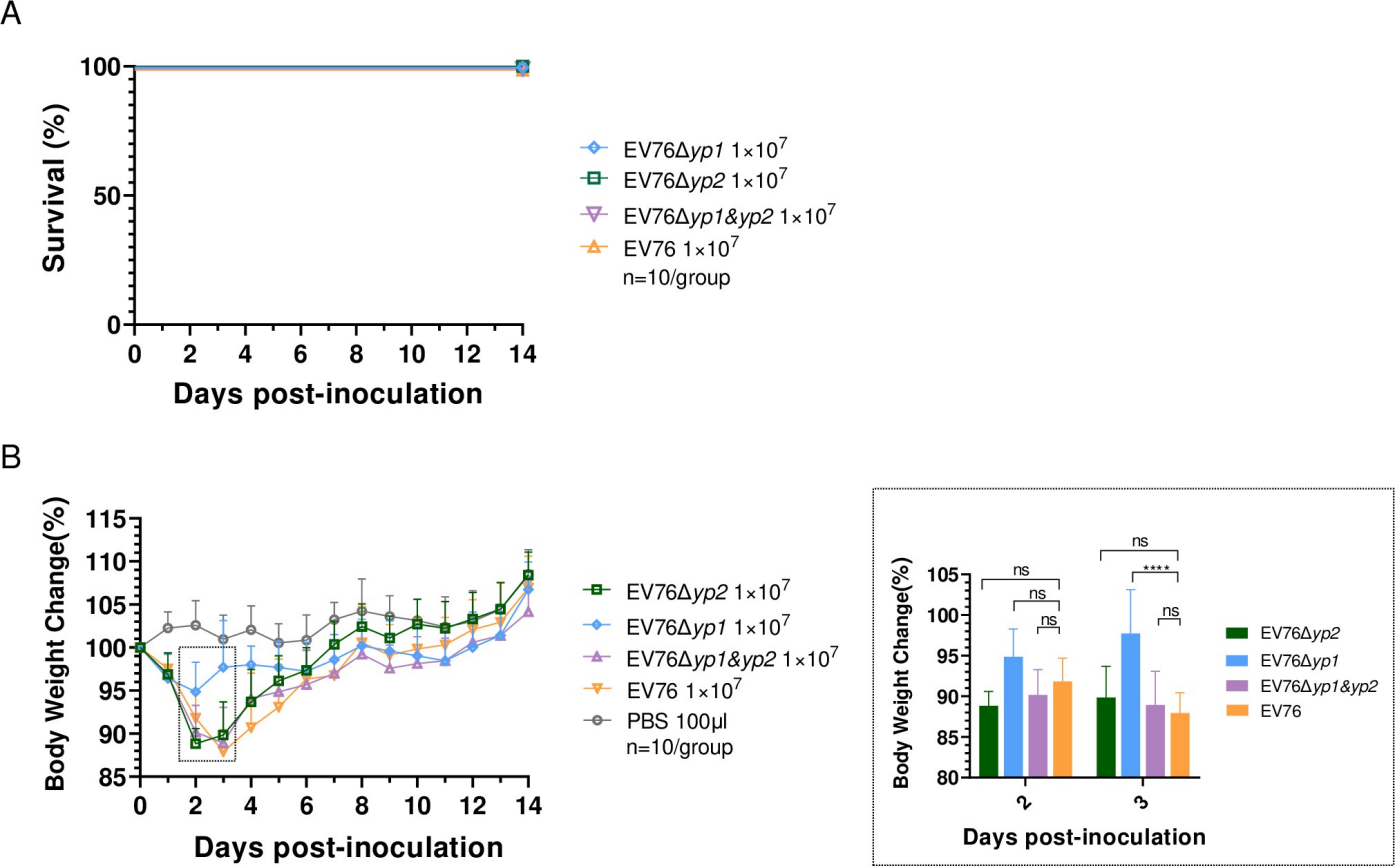
The survival curves and body weight loss of mice *s*.*c* inoculated with the EV76-derived candidate vaccines. **(A)** BALB/c mice (n = 10, female) were inoculated *s*.*c*. with 1×10^7^ CFU of *Y*. *pestis* EV76, EV76Δ*yp1*, EV76Δ*yp2* or EV76Δ*yp1&yp2*, respectively; **(B)** Body weight was monitored continuously for 14 days. The weight changes on days 2 and 3 were shown in detail in the right panel. One-way ANOVA with Tukey *post hoc* test was used to analyze the significance of differences in body weight losses among the infected groups. ns indicates no statistical significance. ****P<0.0001.

Previous studies showed that LD_50_ of EV76 in mice challenged subcutaneously was determined to be 6.3×10^7^ CFU [37]. Therefore, we administered a subcutaneous inoculation of 1×10^7^ CFU of EV76, EV76Δ*yp2*, EV76Δ*yp1*, or EV76Δ*yp1&yp2*, respectively, to groups of BALB/c mice, and monitored their survival and weight for 14 days to assess their residual virulence. All the mice survived after inoculation with the four strains (Fig 1A). The body weight of mice inoculated with EV76Δ*yp1* or EV76Δ*yp2* strain reached its lowest level at 2 days post-inoculation (dpi.), displaying average levels of 94.85% and 88.83% of their body weight before inoculation, respectively. Mice inoculated with the EV76 or EV76Δ*yp1&yp2* strain experienced the lowest body weight at 3 dpi., with an average of 87.92% and 88.91% of their initial body weight, respectively. At 3 to 4 dpi., the body weight of mice began to recover, and the subsequent trend of body weight increase in the four immunized groups became similar after 6 dpi. (Fig 1B). In summary, inoculation with EV76Δ*yp1* had significantly lower impact on the body weight of mice, whereas EV76Δ*yp2* and EV76Δ*yp1&yp2* strains exhibited comparable effects to EV76.

It has been reported that attenuated *Y*. *pestis pgm*^-^ strains have the ability to regain virulence under conditions of iron overload [31,32]. Thus, we further assessed the virulence of the mutant strains in mice with iron overload. BALB/c mice (n = 5 per group) were administered 100 μg of FeCl_2_ and subsequently *s*.*c*. inoculated with ~10^7^ CFU of EV76, EV76Δ*yp2*, EV76Δ*yp1* or EV76Δ*yp1&yp2*. After inoculation, the mice received daily injections of 100 μg of FeCl_2_ and were consecutively monitored for 14 days.

**Fig 2:**
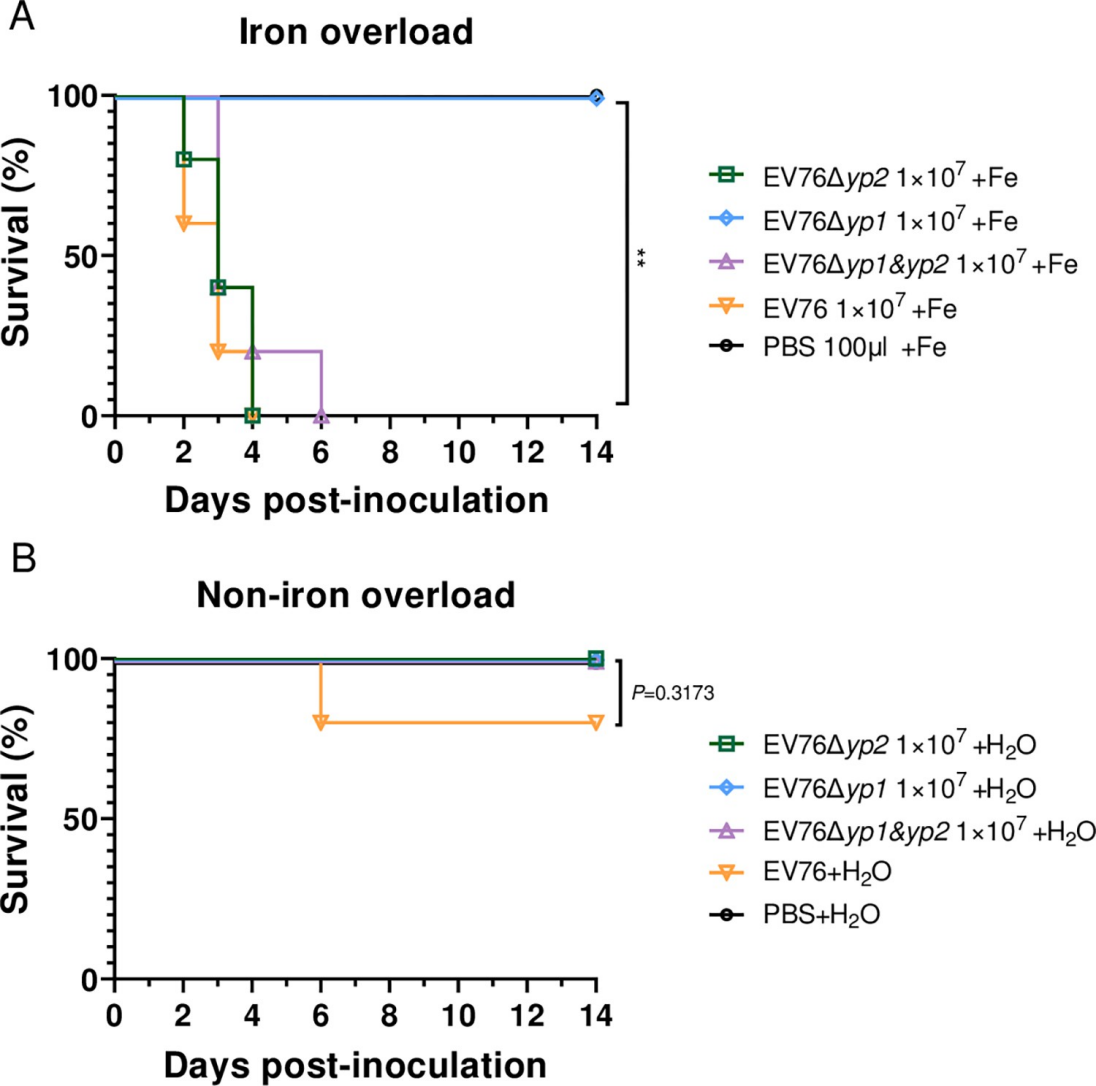
The survival curves of mice inoculated with the EV76-derived candidate vaccines under iron overload conditions. BALB/c mice (n = 5 per group, females) were inoculated *s*.*c*. with 1×10^7^ CFU of EV76, EV76Δ*yp2*, EV76Δ*yp1* or EV76Δ*yp1&yp2*, respectively, and subsequently intraperitoneally (*i*.*p*.) injected daily with 100 μg of FeCl_2_ **(A)** or sterile PBS **(B).** Kaplan–Meier analysis with log-rank (Mantel-Cox) test was used to calculate *P* values, comparing the results to the EV76-inoculated groups. **P<0.01.

As shown in the survival curves, all mice inoculated with EV76Δ*yp1* survived, while those inoculated with EV76Δ*yp2*, EV76Δ*yp1&yp2*, and EV76 succumbed between 2 and 6 dpi. under iron overload conditions (Fig 2A). With the exception of one mouse that died after being inoculated with EV76, all the mice in the non-iron overload groups survived challenges with EV76 and the 3 mutants (Fig 2B). This indicates that EV76Δ*yp1* exhibited significantly better safety compared to EV76 in iron overload mice. However, no improvement was observed for EV76Δ*yp2* and EV76Δ*yp1&yp2*.

**Fig 3:**
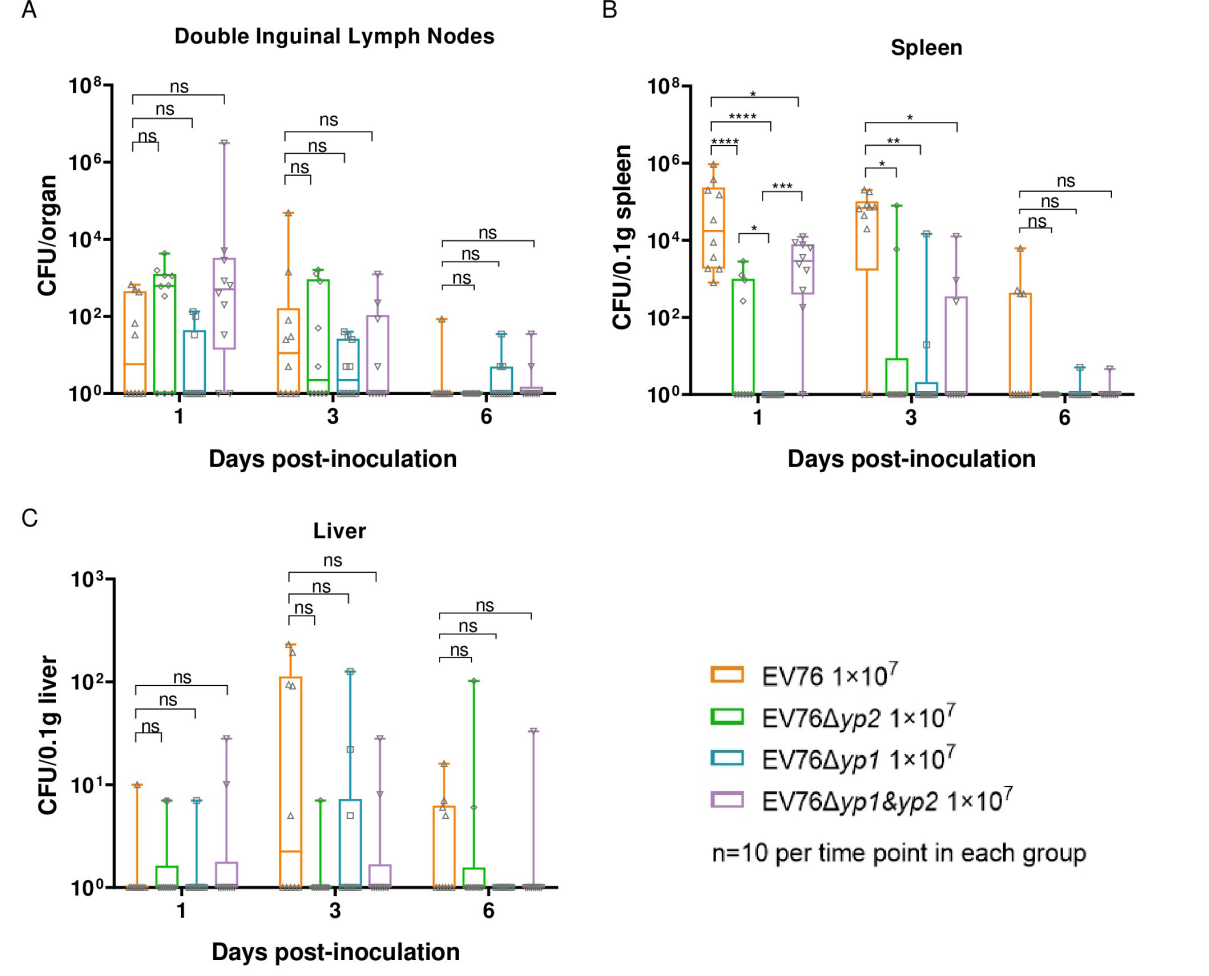
Bacterial loads in organs of mice inoculated with the EV76-derived candidate vaccines. Mice (n = 10 per time point in each group) were inoculated *s*.*c*. with 1×10^7^ CFU of EV76, EV76Δ*yp2*, EV76Δ*yp1*, or EV76Δ*yp1&yp2*. At different time points, mice were sacrificed, and bilateral inguinal lymph nodes **(A)**, spleens **(B)** and liver **(C)** were collected, and the number of living bacteria was determined. Two-way ANOVA with Tukey’s *post hoc* test was used to calculate significant differences in bacterial loads. ns indicates no statistical significance, *P<0.05, **P<0.01, ***P<0.001, ****P<0.0001.

To further evaluate the residual virulence of mutant strains, we analyzed the bacterial loads in the lymph nodes, spleens, and liver of mice that were inoculated *s*.*c*. with approximately 1×10^7^ CFU of EV76, EV76Δ*yp2*, EV76Δ*yp1*, or EV76Δ*yp1&yp2* at bilateral groins, using equal amounts of bacterial suspensions. At 1, 3, and 6 dpi., animals (n = 10 per group) were sacrificed, and their inguinal lymph nodes, spleen, and liver were harvested for bacterial load determination. The results showed that there was no significant difference in the bacterial load in the inguinal lymph nodes and liver among the four groups across all the time points (Fig 3A and 3C).

Interestingly, the bacterial loads in the spleen of mice inoculated with the three mutant strains were significantly lower than those in mice inoculated with EV76 at 1 and 3 dpi. (Fig 3B), suggesting a faster clearance of the mutant strains. These results indicate that all three mutants exhibited similar efficiency as EV76 in traveling to draining lymph nodes, but they were cleared more rapidly from the spleen. Moreover, there was no significant difference in the distribution of the three mutant strains to the liver compared to EV76.

**Fig 4:**
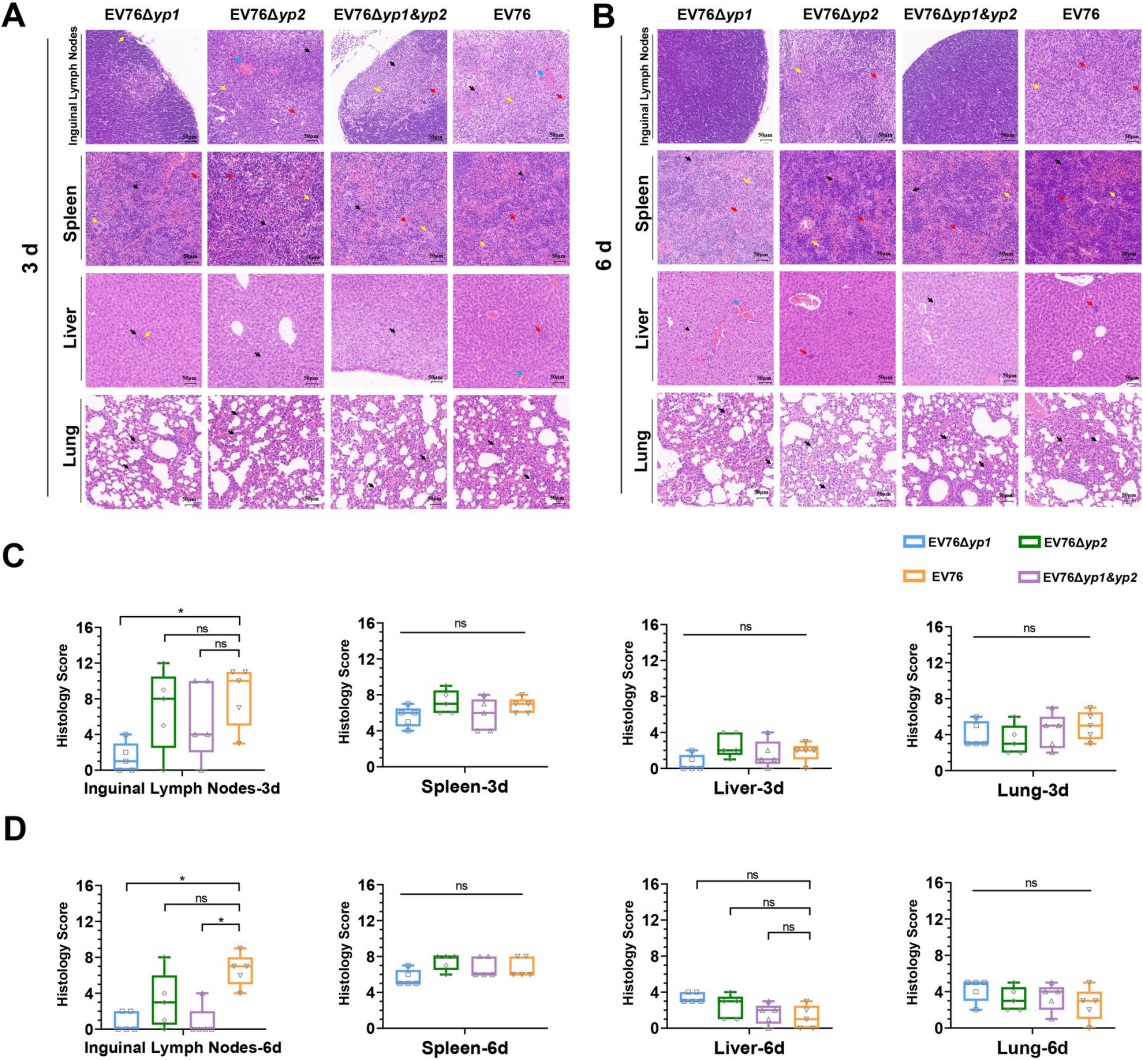
Histopathological analysis on mouse tissues following inoculation with the EV76-derived candidate vaccines. Mice (n = 5 per time point in each group) were inoculated *s*.*c*. with 1×10^7^ CFU EV76, EV76Δ*yp2*, EV76Δ*yp1*, or EV76Δ*yp1&yp2*. Representative hematoxylin-eosin (HE) staining results for the different tissues at 3 dpi. **(A)** and 6 dpi. **(B)** are shown as indicated. HE staining of the lymph nodes tissue sections revealed various degrees of inflammatory cell infiltration (red arrows), necrosis (black arrows), connective tissue hyperplasia (yellow arrows), and congestion (blue arrows). The spleen tissue sections showed different degrees of inflammatory cell infiltration (red arrows), massive extramedullary hematopoiesis (black arrows), and a mild increase of multinucleated giant cells (yellow arrows). The liver tissue sections showed degeneration of hepatocytes (black arrows), few foci of extramedullary hematopoiesis (red arrows), and few venous congestion and vasodilatation (blue arrows), inflammatory cell infiltration (yellow arrows). Pathological examination of lung tissues from mice revealed mild inflammatory cell infiltration (black arrows), with limited thickening of alveolar walls observed. The histopathological scores of various organs at **(C)** 3 dpi. and **(D)** 6 dpi. were determined using Kruskal-Wallis with Dunn’s *post hoc* test to assess statistical significance. *P<0.05.

We also performed histopathological analysis on various tissues from mice inoculated *s*.*c*. with 1×10^7^ CFU of EV76, EV76Δ*yp2*, EV76Δ*yp1*, or EV76Δ*yp1&yp2*. At 3 or 6 dpi., the mice were sacrificed, and the inguinal lymph nodes, spleens, lungs, and livers were harvested for examination of pathological alterations (Fig 4A and 4B). At 3 dpi., inflammatory lesions were observed in lymph nodes, spleen, liver, and lungs of all the inoculated groups. The pathological scores in the inguinal lymph nodes of the EV76Δ*yp1*-inoculated group were lower compared to the other groups, while no statistically significant difference was observed in the spleen, liver, and lungs among the groups (Fig 4C). At 6 dpi., the pathological score of inguinal lymph nodes in the EV76Δ*yp1*- and EV76Δ*yp1&yp2*-inoculated group was lower than that of EV76- and EV76Δ*yp2*-inoculated group. The lymph nodes in the former two groups returned to normal size, while those in the latter two groups still exhibited pathological changes, such as inflammatory cell infiltration and connective tissue hyperplasia (Fig 4D). The deletion of the *yp1* gene from EV76 significantly reduces damage to draining lymph nodes. Taken together, these results demonstrate that EV76Δ*yp2*, EV76Δ*yp1*, and EV76Δ*yp1&yp2* exhibit varying degrees of reduced virulence compared to EV76, thus they are promising candidate for further evaluation as attenuated living vaccines against plague.

### Protection efficacy of EV76-derived candidate vaccines in short-term study

Mice were immunized *s*.*c*. twice at a 21-day interval with a dose of 5×10^6^ CFU of EV76, EV76Δ*yp2*, EV76Δ*yp1* or EV76Δ*yp1&yp2*. The evaluation of humoral and cellular immune responses, as well as the protective effect of immunization against the virulent *Y*. *pestis* strain, was performed six weeks after initial immunization.

#### 1. Evaluation of the specific humoral immune response in mice immunized with candidate EV76-derived vaccines

**Fig 5:**
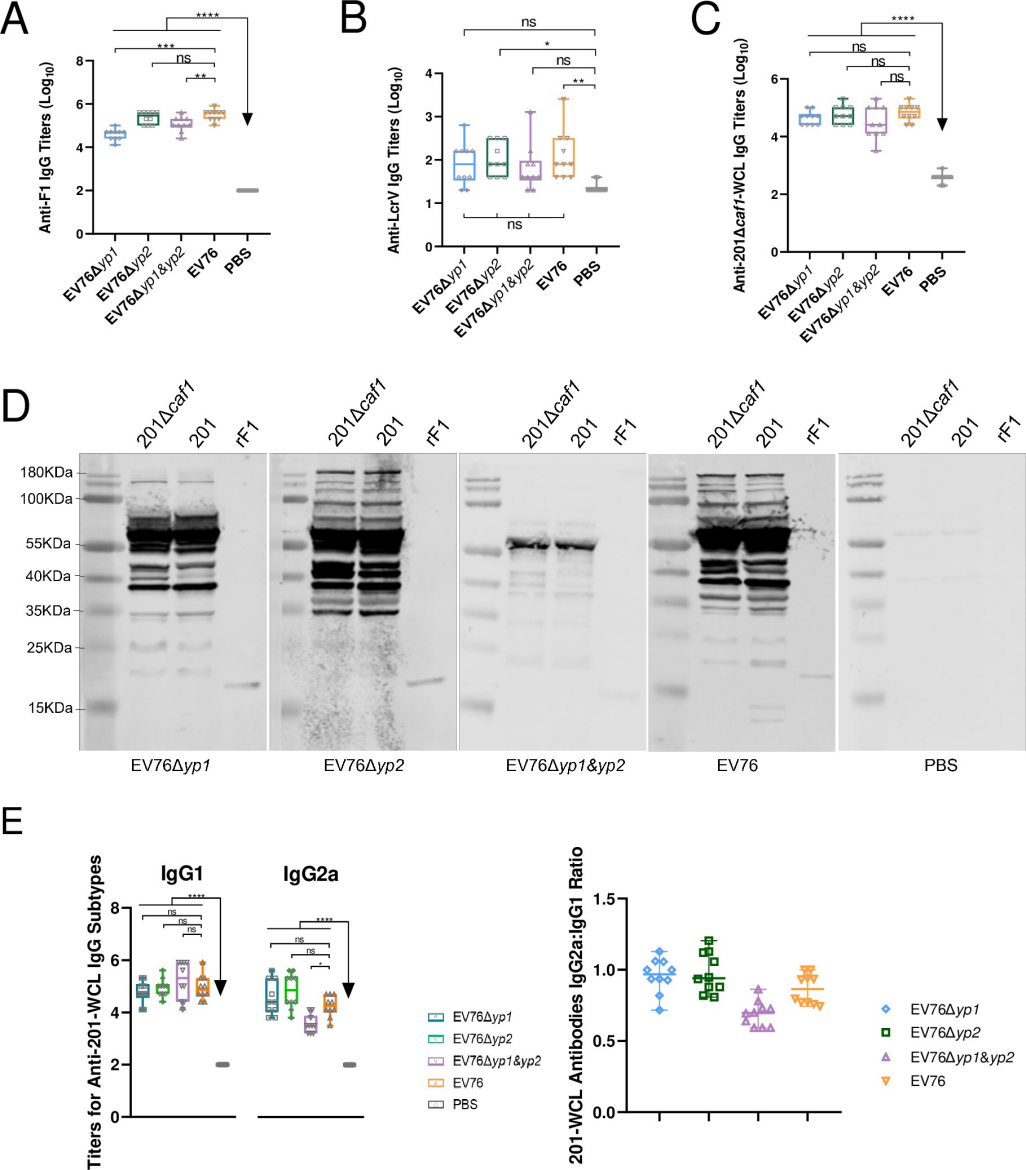
Humoral immune responses in mice administrated *s*.*c*. with the EV76-derived candidate vaccines in short-term study. Mice (n = 10 per group) were immunized *s*.*c*. with two doses of 5×10^6^ CFU of EV76, EV76Δ*yp2*, EV76Δ*yp1* or EV76Δ*yp1&yp2* at a 21-day interval. Sera were collected on day 41 after the initial immunization. Titers of IgG specific to F1 **(A),** LcrV **(B),** and 201Δ*caf1*-WCL **(C)** were measured using ELISA. **(D)** For Western blotting analysis, blotted antigens were obtained from the whole cell lysates of 201 or 201Δ*caf1*, and pooled sera from immunized mice were used as primary antibodies. **(E)** IgG subclasses to 201-WCL antigen were analyzed by ELISA. (**F**) The serum IgG2a/IgG1 ratios in the immunized mouse groups were determined. Statistical analysis was conducted using One-way ANOVA with Tukey *post hoc* to determine the significance of differences. *P<0.05, **P<0.01, ***P<0.001 and ****P<0.0001.

Sera were collected from vaccinated mice 41 days after initial immunization. The levels of serum IgG titers against antigens F1 and LcrV were measured using ELISA. Among all the three EV76 mutants, all of them induced significant levels of IgG titer against F1 antigen in *s*.*c*. immunized mice (Fig 5A). However, only EV76Δ*yp2*, but not EV76Δ*yp1* and EV76Δ*yp1&yp2*, elicited IgG titers against F1 comparable to those induced by EV76 in mice. None of the tested attenuated strains induced high IgG titer against LcrV antigen (Fig 5B), although EV76Δ*yp2-* and EV76-immunized groups showed slightly higher IgG titers against LcrV compared to the control group.

To assess the IgG titer against antigens other than F1, the serum IgG titer against the whole cell lysate of *Y*. *pestis* 201Δ*caf1* (201Δ*caf1-*WCL) was measured. The results showed that all three EV76 mutants induced significantly higher levels of IgG against 201Δ*caf1*-WCL in mice compared to the group treated with PBS (phosphate buffer saline) alone (Fig 5C). Consistently, Western blot analysis confirmed that besides F1 antigen, immune sera also recognized multiple antigens in 201Δ*caf1-*WCL (Fig 5D). Immunoblotting analysis of serum samples from the EV76Δ*yp2*- and EV76-immunized mice revealed similar protein patterns, while some bands were missing for serum samples from the EV76Δ*yp1* and EV76Δ*yp1&yp2*-immunized groups, suggesting that the deletion of the *yp1* gene might impact the expression or immunoactivities of certain antigens.

The IgG1 and IgG2a subclasses of serum IgG against 201-WCL were analyzed to provide further insight. We observed significantly higher titers of IgG1 and IgG2a against 201-WCL in all the immunized mice compared to the control group (Fig 5E). Among the strains tested, all except EV76Δ*yp1&yp2* induced comparable IgG2a titers against 201-WCL as observed in the EV76-immunized group. Regarding the IgG1 subclasses of anti-201-WCL IgG, no significant differences were observed between the different groups.

The anti-201-WCL IgG2a/IgG1 ratios in the EV76Δ*yp1*-, EV76Δ*yp2*-, EV76-immunized groups were approximately to 1.0 (Fig 5F), suggesting the induction of a balanced Th1/Th2 immune response by these three strains. In contrast, the EV76Δ*yp1&yp2*-immunized group displayed an anti-201-WCL IgG2a/IgG1 ratio of 0.69 (Fig 5F), pointing to a Th2-skewed response.

#### 2. Characterizing specific cellular immune response in mice immunized with EV76-derived candidate vaccines

To assess cell-mediated immune responses in vaccinated mice, splenocytes were isolated and stimulated *in vitro* with the whole cell lysate of *Y*. *pestis* 201 (201-WCL) acquired by sonication. The cytokine levels in the cell culture supernatants were measured using a cytokines determination by Luminex assay (n = 5 per group). The EV76Δ*yp1*-immunized group was excluded from the analysis due to its inferior performance in humoral immunity.

**Fig 6:**
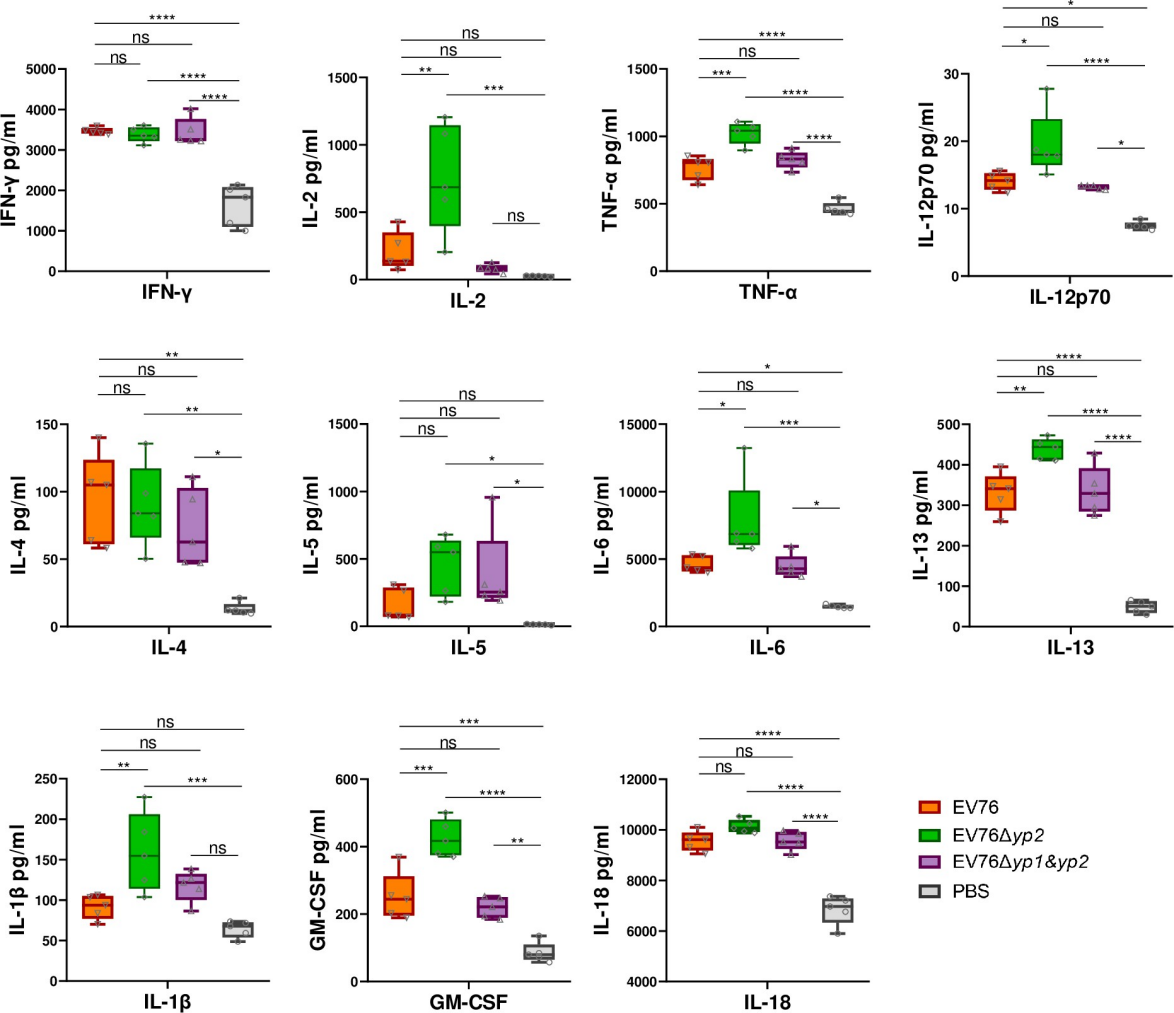
Evaluation of cellular immune responses in mice inoculated *s*.*c*. with EV76-derived candidate vaccines. Splenocytes were harvested and stimulated with 201-WCL and the supernatants were evaluated for cytokines levels by Luminex assay (n = 5 for each group). One-way ANOVA with Tukey *post hoc* was used to determine the significance of differences. *P<0.05, **P<0.01, ***P<0.001, ****P<0.0001.

When stimulated with 201-WCL, splenocytes from mice immunized with EV76Δ*yp2* secreted significantly higher levels of cytokines, including IL-12p70, IL-13, IL-1β, IL-2, IL-6, TNF-α, and GM-CSF, compared to splenocytes from mice immunized with EV76 (Fig 6). In contrast, no discernible difference was observed in cytokine secretions between the EV76Δ*yp1&yp2*-immunized and the EV76-immunized groups. Similar results were observed when splenocytes were stimulated with 201Δ*caf1-*WCL (S3 Fig), suggesting that vaccination with EV76 and the mutants elicited similar cell-mediated immune responses against *Y*. *pestis* 201Δ*caf1* and 201.

These results indicated that EV76Δ*yp2* elicits a stronger cell-mediated immune compared to EV76, indicating its potential to provide enhanced protection against plague. In addition, this increased response induced by EV76Δ*yp2* was observed when the splenocytes were stimulated with both 201-WCL and 201Δ*caf1*-WCL (S3 Fig), suggesting the involvement of multiple antigens beyond F1 in the cell-mediated immune responses against plague following immunizations.

#### 3. Protective efficacy of candidate vaccines derived from EV76 in immunized mice

To enable further investigation into the protective efficacy, we assessed the susceptibility of BALB/c mice to 201, 201Δ*caf1* or 201-*lux*. The result was shown in S3 Table.

**Fig 7:**
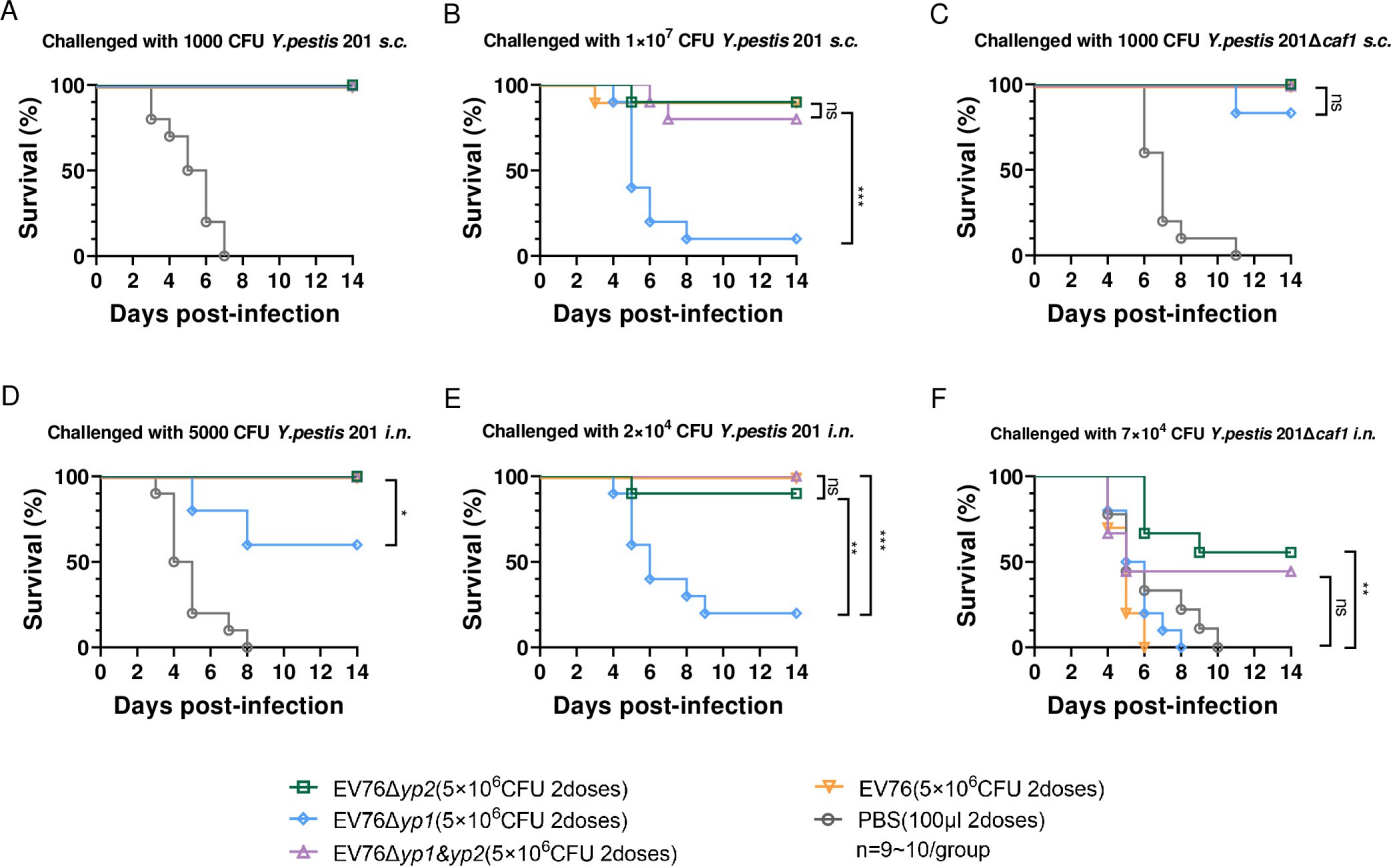
Efficacy of EV76-derived candidate vaccines in protecting immunized mice against exposure to *Y*. *pestis* 201 or 201Δ*caf1*. Mice were immunized *s*.*c*. twice at 21-day interval with 5×10^6^ CFU of EV76, EV76Δ*yp2*, EV76Δ*yp1*, or EV76Δ*yp1&yp2*, respectively. Vaccinated mice were subjected to *s*.*c*. or *i*.*n*. challenge 42 days after the initial immunization. Groups of mice injected with PBS instead of the bacterial suspension served as a control. Vaccinated mice (n = 9~10 per group) were challenged *s*.*c*. with 1000 CFU (322 LD_50_) **(A)**, 1×10^7^ CFU (3.22×10^6^ LD_50_) **(B)** of *Y*. *pestis* 201, 1000 CFU (403 LD_50_) of *Y*. *pestis* 201Δ*caf1* **(C)**, or *i*.*n*. challenged with 5000 CFU (11.4 LD_50_) **(D)**, 2×10^4^ CFU (45.6 LD_50_) **(E)** of *Y*. *pestis* 201, or challenged *i*.*n*. with 7×10^4^ CFU (207 LD_50_) of *Y*. *pestis* 201Δ*caf1* **(F)**. Kaplan–Meier analysis with log-rank (Mantel-Cox) test was used to determine the significance of differences between the survival curves. ns, no significance, *P<0.05, **P<0.01, ***P<0.001.

On day 42 after the initial immunization, vaccinated mice were subjected to *s*.*c*. or *i*.*n*. challenges with *Y*. *pestis* 201. All mice in the immunized group survived when exposed to a low dose of 1000 CFU (322 LD_50_) of *Y*. *pestis* 201 (Fig 7A) and no symptoms such as ruffled fur, hunch back or lethargy were observed. In contrast, all the unvaccinated mice in the control group succumbed to death following the same challenges. When challenged with a high dose of 1×10^7^ CFU (3.22×10^6^ LD_50_) of *Y*. *pestis* 201, 90% of the mice in both the EV76- and EV76Δ*yp2*-immunized group survived, and 80% of the mice in the EV76Δ*yp1&yp2*-immunized group survived. In contrast, only 10% of the mice in EV76Δ*yp1*-immunized group survived this challenge (Fig 7B).

The immunization with EV76, EV76Δ*yp2*, or EV76Δ*yp1&yp2* provided mice with complete protection against *i*.*n*. challenge with 5000 CFU (11.4 LD_50_) of *Y*. *pestis* 201. In contrast, immunization with EV76Δ*yp1* conferred only 60% protection to the mice (Fig 7D). After being challenged *i*.*n*. with 2×10^4^ CFU (45.6 LD_50_) of *Y*. *pestis* 201, all the mice in the EV76- and EV76Δ*yp1&yp2*-immunized group survived, 90% of the mice in the EV76Δ*yp2*-immunized group survived, and only 20% of the mice in EV76Δ*yp1*-immunized group survived (Fig 7E).

To evaluate the protective efficacy of these EV76-derived candidate vaccines against non-encapsulated *Y*. *pestis*, mice were immunized *s*.*c*. twice at a 21-day interval with 5×10^6^ CFU of EV76, EV76Δ*yp2*, EV76Δ*yp1* or EV76Δ*yp1&yp2*. On day 42 after the initial immunization, the vaccinated mice were subjected to *s*.*c* or *i*.*n*. challenges with virulent *Y*. *pestis* 201Δ*caf1*. The immunization with EV76, EV76Δ*yp2*, or EV76Δ*yp1&yp2* provided mice with complete protection against *s*.*c*. challenge with 1000 CFU (403 LD_50_) of *Y*. *pestis* 201Δ*caf1*, whereas immunization with EV76Δ*yp1* conferred only 80% protection to the mice (Fig 7C). After being challenged *i*.*n*. with 7×10^4^ CFU (207 LD_50_) of *Y*. *pestis* 201Δ*caf1*, 55.6% of the EV76Δ*yp2*-immunized mice and 44.4% of the EV76Δ*yp1&yp2*-immunized mice survived. By contrast, none of mice in the EV76Δ*yp1*- and EV76-immunized group survived (Fig 7F), suggesting that EV76Δ*yp2* provides superior protective efficacy against non-encapsulated *Y*. *pestis* compared to EV76.

### Rapid protection provided by a single-dose immunization of EV76-derived candidate vaccines

**Fig 8:**
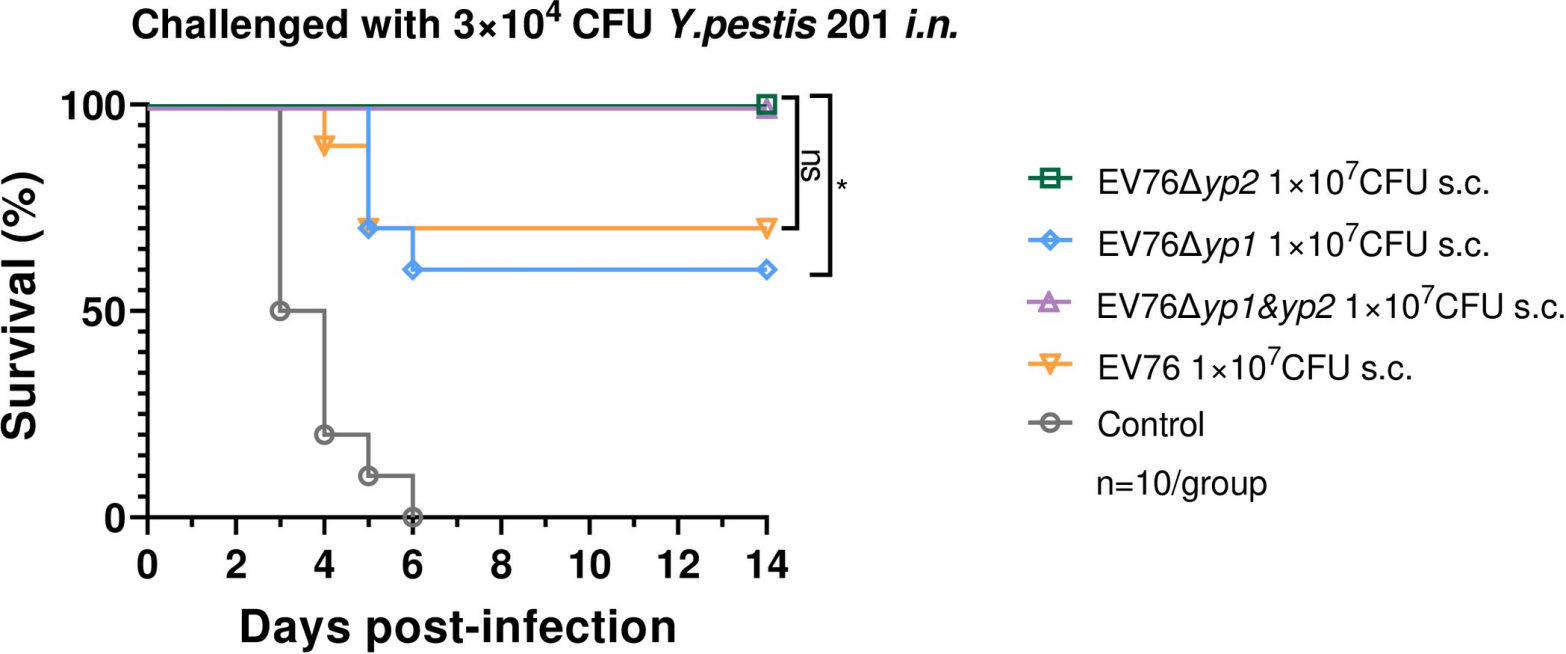
Evaluation of rapid protection efficacy in mice immunized with a single-dose of EV76-derived candidate vaccines. Mice (n = 10 per group) were immunized *s*.*c*. with 1×10^7^ CFU of EV76, EV76Δ*yp2*, EV76Δ*yp1*, or EV76Δ*yp1&yp2*. After 21 days, the vaccinated mice were subjected to *i*.*n*. challenge with 3×10^4^ CFU (68LD_50_) of *Y*. *pestis* 201. Kaplan–Meier analysis with log-rank (Mantel-Cox) test was used to determine the significance of differences. ns, no significance, *P<0.05.

As previously reported, live-attenuated plague vaccines have been shown to provide rapid protection against *Y*. *pestis* with a single-dose immunization [23,38], making them particularly suitable for emergency use in the face of imminent risk of exposure. To assess the ability of different EV76-derived candidate vaccines to confer rapid protection through a single-dose immunization, mice were immunized *s*.*c*. with 1×10^7^ CFU of EV76, EV76Δ*yp2*, EV76Δ*yp1*, or EV76Δ*yp1&yp2*. After 21 days, the mice were subjected to *i*.*n*. challenge with *Y*. *pestis* 201 to evaluate the protective efficacy of the vaccines. The immunization with EV76Δ*yp2*, or EV76Δ*yp1&yp2* provided the mice with complete protection against *i*.*n*. challenge of 3×10^4^ CFU (68 LD_50_) of *Y*. *pestis* 201. However, the immunization with EV76Δ*yp1* and EV76 only resulted in a 60% and 70% of mice survival rate, respectively (Fig 8).

### Protection efficacy of EV76-derived candidate vaccines in long-term study

**Fig 9:**
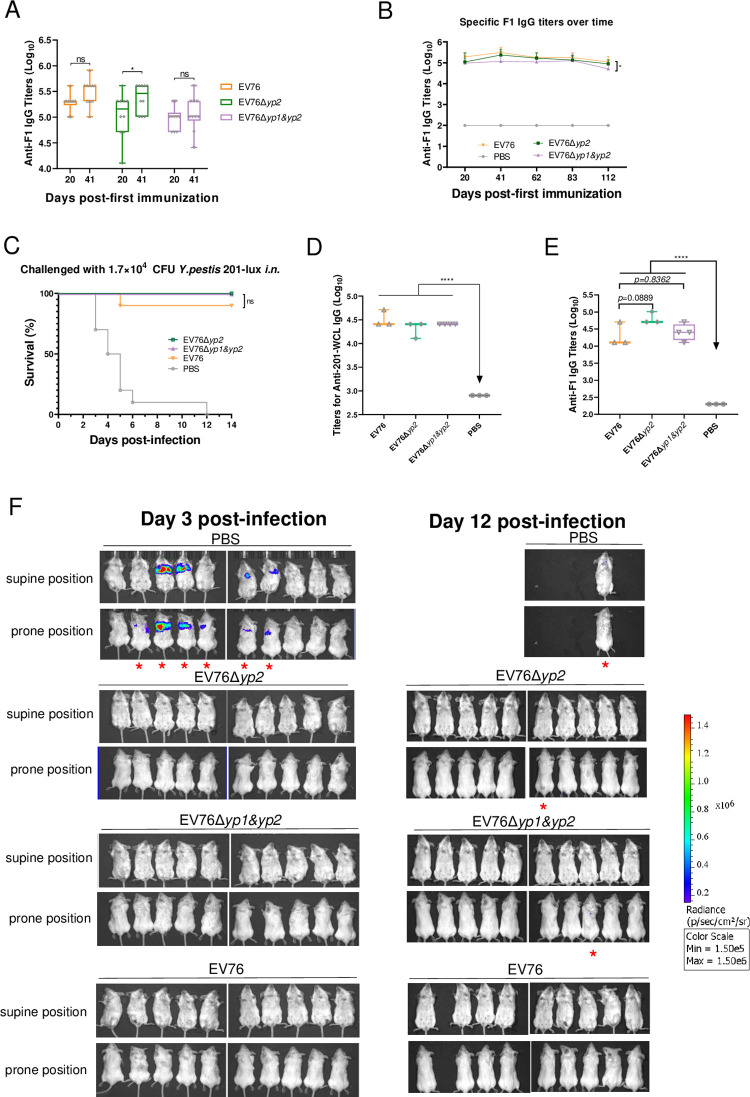
Efficacy of EV76-derived candidate vaccines in protecting mice from *i*.*n*. challenge with *Y*. *pestis* 201-*lux*. Mice (n = 10 each group) were immunized *s*.*c*. twice at a 21-day interval with 5×10^6^ CFU either of EV76, EV76Δ*yp2*, EV76Δ*yp1&yp2*, or PBS. The IgG titers against F1 antigen in the sera of mice were then determined by ELISA. **(A)** The IgG titers against F1 after initial and booster immunization. **(B)** The kinetics of IgG titers against F1 were monitored over 112 days. Two-way ANOVA with Tukey *post hoc* was used to determine the significance of differences in IgG titers between the different groups **(C)** On day 120 post initial-immunization, the vaccinated mice were challenged *i*.*n*. with 1.7×10^4^ CFU (19.7 LD_50_) of *Y*. *pestis* 201-*lux*. Mouse survival was monitored daily for 14 days and the survival curves were plotted using GraphPad 8.0.1 Kaplan–Meier analysis with log-rank (Mantel-Cox) test was used to determine the significance of differences. One-year post initial immunization, IgG titers against 201-WCL **(D)** or F1 antigen **(E)** were measured in the sera of mice (n = 3 or 4 per group) immunized with the indicated *Y*. *pestis* strains. One-way ANOVA with Dunnett was used to determine the significance of differences. **(F)** In vivo luminescence imaging of mice *i*.*n*. challenged with *Y*. *pestis* 201-*lux*. On 3 and 12 dpi, surviving mice were imaged, and those exhibiting luminescence signals were denoted with red asterisks. The luminescent intensity ranged from 1.5×e^6^ (red) to 1.5×e^5^ (violet) as indicated. ns, no significance, *P<0.05, **** *P<*0.0001.

To evaluate the long-term protection efficacy of these attenuated mutants, mice were immunized *s*.*c*. twice at a 21-day interval with 5×10^6^ CFU of EV76, EV76Δ*yp2*, or EV76Δ*yp1&yp2*. Serum samples were collected from the mice at days 20, 41, 62, 83, and 112 after the initial immunization, and specific IgG titers were determined by ELISA analysis. At day 20 post-initial immunization, the immunized mice in all three groups demonstrated elevated IgG titers against F1 antigen. Specifically, the EV76Δ*yp2*-immunized group exhibited a significant increase in F1 IgG titers following the booster immunization compared to the initial immunization (Fig 9A). However, no significant difference was found between the initial and the booster immunization in mice vaccinated with EV76Δ*yp1&yp2* or EV76. Following two doses of *s*.*c*. inoculations, the IgG titers in all three groups reached their highest levels at day 41 post-initial immunization. Subsequently, the titers gradually declined but remained consistently high, ranging from 35% to 37% of the maximum IgG titers, during a period of 112 days post-initial immunization (Fig 9B). Among the mice immunized with the two-dose inoculation, a group of mice (n = 3–4 for each candidate vaccines) were left untreated, and their sera were collected one year after the initial immunization to measure the IgG titers against 201-WCL or F1 antibody. As depicted in Fig 9D and 9E, immunized mice in all three groups exhibited high IgG titers against 201-WCL or F1 antigen one year after the initial immunization. Interestingly, the mean titers against F1 antibody in mice immunized with EV76Δ*yp2* were found to be higher than those in mice immunized with EV76, although the difference was not statistically significant (*p* = 0.0889). This observation suggests that the presence of F1 antibody may persist for a longer duration in EV76Δ*yp2*-immunized mice compared to that in EV76-immunized mice.

At day 120 after initial immunization, mice were challenged *i*.*n* with 1.7×10^4^ CFU (19.7 LD_50_) of *Y*. *pestis* 201-*lux*, which express luciferase and its substrates [39]. The immunization with EV76Δ*yp2* and EV76Δ*yp1&yp2* provided complete protection to mice against *i*.*n*. challenge with *Y*. *pestis* 201-*lux*, whereas 10% of EV76-immunized group succumbed to the infection (Fig 9C). Mice were imaged by IVIS (In Vivo Imaging System) to track the dissemination of *Y*. *pestis* 201-*lux* during the infection. At 3 dpi., out of the 10 mice in the control group injected with PBS, 7 exhibited persistent luminescence signals in the lungs, whereas no luminescence signal was detected in all the vaccinated mice. In the control group, 90% of mice (9 out of 10) succumbed to the infection prior to 12 dpi. The only surviving mouse exhibited luminescence around the trachea, along with symptoms such as hair shrugging and significant weight loss. The mouse expired shortly after undergoing imaging. In the EV76-immunized group, one mouse succumbed to the infection before imaging at 12 dpi., and no luminescence signal was detected in the remaining mice. In the EV76Δ*yp2*- and EV76Δ*yp1&yp2*-immunized group, all mice survived, with only one mouse in each group exhibiting weak and limited luminescence signals (Fig 9F). These results suggest that protection provided by EV76Δ*yp2* or EV76Δ*yp1&yp2* is comparable to EV76 at 120 days post-vaccination.

## Discussion

Vaccine candidate should provide significant protection with minimal side effects. The live-attenuated vaccine EV76 has been used in tens of millions of people since 1936 and provides protection against both bubonic and pneumonic plague. However, its variable lethality in some animal models [40,41] and reactogenicity in humans have hindered its global acceptance [42]. Nevertheless, modification of different virulence associated genes, i.e., *lpxM* or *pla* [37,43], has improved safety while retaining its immunogenicity. In this study, we investigated whether deletion of the sORF gene *yp1* and *yp2*, based on the EV76 vaccine strain, hold the promise of creating a more potent and safer live attenuated plague vaccine.

The significant attenuation of virulence in the EV76Δ*yp1* strain was evidenced by a number of experimental observations. These included a markedly reduced loss of body weight in mice (Fig 1B), avirulence under iron overload conditions (Fig 2A), a significantly lower bacterial burden in the spleen (Fig 3B) and reduced pathological damage to the local lymph nodes when compared to EV76. The bacterial loads of EV76Δ*yp2* and EV76Δ*yp1&yp2* in the spleen were significantly reduced compared to EV76 at both 1 or 3 dpi. (Fig 3B). Although no difference was found between them and EV76 in other animal experiments, these results suggest that the two strains exhibit lower virulence than EV76 in terms of dissemination. Intriguingly, the double gene mutant strain EV76Δ*yp1&yp2* did not exhibit the virulence attenuation observed in EV76Δ*yp1*. This suggests a complex interaction between *yp1* and *yp2* genes that may underlie this phenomenon, which needs further exploration.

Previous studies have demonstrated that live-attenuated vaccines against *Y*. *pestis* can effectively trigger both humoral and cell-mediated immune responses [43–46]. It has also been established that F1 and LcrV are the most important protective antigens [30,47]. Our findings demonstrated that immunization with the various EV76-derived candidate vaccines resulted in remarkably elevated serum IgG titers against F1 in mice (Fig 5A). However, the induction on IgG titers against LcrV was negligible, with a significantly higher level observed only in mice immunized with EV76Δ*yp2* or EV76, but not in the other groups compared to the control group (Fig 5B). Our observations are consistent with previous studies indicating that live-attenuated vaccines against *Y*. *pestis* have a limited capacity to stimulate an anti-LcrV antibody response. [26,46,48,49]. This finding raises concerns regarding the protective efficacy of live-attenuated vaccines against non-encapsulated *Y*. *pestis* strains. Therefore, we further detected the serum IgG titers and antibody profile against 201Δ*caf1*-WCL (F1 negative). As shown in Fig 5C and 5D, all the vaccinated mice displayed high IgG titers against 201Δ*caf1*-WCL. Moreover, Western blotting analysis revealed that the immune sera recognized a variety of antigens, in addition to F1, indicating a broad antibody response. Considering the potential loss of conformational epitopes during the detection processes, it is plausible that the actual number of antigens successfully recognized by the immune sera was even higher [50].

Previous studies have suggested that *s*.*c*. immunization of attenuated living vaccines against plague tend to favor Th2-biased humoral immune responses [44,51], while intramuscular immunization tend to promote a Th1/Th2 balanced humoral immune response. Our results revealed that *s*.*c*. immunization of EV76Δ*yp1*, EV76Δ*yp2* or EV76 elicited a balanced Th1/Th2 immune response against 201-WCL with IgG2a/IgG1 ratios approximately to 1.0 (Fig 5F), whereas mice immunized with EV76Δ*yp1&yp2* displayed an anti-201-WCL IgG2a/IgG1 ratio of 0.69 (Fig 5F), pointing to a Th2-skewed response. The discrepancy between our observations and the previous finding may be due to the intrinsic characteristics of the specific strains and further experiments are needed to verify this issue.

The bacterial cell lysates of both 201 and 201Δ*caf1* were capable of stimulating splenocytes from immunized mice to secret multiple cytokines including IFN-γ, TNF-α, IL-4, IL-13, IL-6, GM-CSF and IL-18 (S3 Fig). These findings suggest that in addition to F1 antigen, a diverse range of other antigens contribute to the humoral and cellular immune response elicited by live-attenuated vaccines against *Y*. *pestis*. Notably, compared to the EV76-immunized group, EV76Δ*yp2*-immunized mice exhibited a significantly higher secretion level of Th1-related cytokines, including TNF-α, IL-2, and IL-12p70 (Fig 6), which have been recognized as crucial factors in conferring protection against plague [52–56]. These results suggested the potential of EV76Δ*yp2* as a promising candidate vaccine for eliciting enhanced cellular immunity against plague.

The evaluation of immune protection in short-term study revealed that EV76Δ*yp2* and EV76Δ*yp1&yp2* showed similar levels of protection efficacy to EV76, whereas EV76Δ*yp1* was relatively less effective, consisting to the facts that F1 antibody titer was lowest in the group immunized with EV76Δ*yp1*. This observation can be attributed to the rapid clearance of EV76Δ*yp1* in mice, which hindered the stimulation of sufficient adaptive immunity [44,57,58]. Almost all mice immunized with candidate vaccines achieved complete protection against *s*.*c*. challenge of non-encapsulated *Y*. *pestis* (Fig 7C). In contrast, only EV76Δ*yp2* or EV76Δ*yp1&yp2* provided partial protection against *i*.*n*. challenge of the same strain, while EV76 and EV76Δ*yp1* immunizations did not confer significant protection (Fig 7F). It has been reported that the non-encapsulated *Y*. *pestis* can evade immune responses elicited by *pgm*- *Y*. *pestis* strains or F1 subunit vaccines in models of pneumonic plague in mice [19,57,59]. In certain potential application scenarios, EV76Δ*yp2* and EV76Δ*yp1&yp2* demonstrate superior protection efficacy than EV76.

The discovery that deleting the *yp2* gene in EV76 led to a significant enhancement in protection against non-encapsulated *Y*. *pestis* strains is truly exciting. Moreover, both EV76Δ*yp2* and EV76Δ*yp1&yp2* provided superior protection against pneumonic plague compared to EV76 after a single-dose of immunization. We hypothesized this was partially due to that deletion of *yp2* enhanced the presentation of T-cells epitopes by host cells, resulting in an elevated cellular immunity. Taken together, these findings suggest that in specific application scenarios, such as when encountering F1-negative *Y*. *pestis* strains, EV76Δ*yp2* and EV76Δ*yp1&yp2* provide superior protection compared to EV76.

In regards of long-term protective efficacy, we observed no significant difference between protective efficacy of EV76 and EV76Δ*yp2*. However, it is worth noting that all the mice in EV76Δ*yp2* immunized group survived, while one mouse in the EV76 immunized group died. Furthermore, the IgG titer for F1 antigen of serum samples from EV76*Δyp2* immunized group was slightly higher than that of the EV76 immunized group, with a *p*-value of 0.089. We speculate that this difference may become statistically significant when the number of the animal (currently n = 3) is increased.

The development of vaccines against various mutant strains is a significant challenge in the field of plague vaccine research. Not only do natural non-encapsulated *Y*. *pestis* strains pose a challenge, but strains expressing LcrV variants are also able to evade the immune protection by LcrV subunit vaccine. These mutants possess the ability to bypass plague vaccines that rely solely on F1 and LcrV for protection [60]. Live-attenuated plague vaccines, with their complex antigenic composition, show potential in providing protection against various virulent *Y*. *pestis* strains. One example is the *yscN* mutant of *Y*. *pestis* C12, a strain lacking the ability to produce the F1 antigen. Although this strain is unable to elicit anti-LcrV antibodies, it still provides partial protection against bubonic plague in 40% of animals *s*.*c*. challenged [46]. This observation suggests that live attenuated vaccines can offer partial protection against bubonic plague, even without the protection provided by F1 and LcrV antigens. Several studies have established the crucial role of LcrV antibodies in protecting against non-encapsulated *Y*. *pestis* [19,61–63]. Unfortunately, live-attenuated vaccines have not been effective in inducing significant production of LcrV antibodies in mice, resulting in their suboptimal performance in protection against non-encapsulated *Y*. *pestis*. On the other hand, cellular immunity is also paramount for protection against plague infection [55,64] since a significant number of *Y*. *pestis* antigens other than F1 and LcrV can also be recognized by T cells [65]. Enhancing the cellular immunity elicited by live-attenuated vaccines towards these antigens other than F1 and LcrV, rather than solely relying on humoral immunity triggered by these antigens, may represent a feasible approach to improving the protective effect of live-attenuated vaccines. Our study highlights that deletion of the *yp2* gene from EV76 did not increase LcrV antibody production, but significantly enhanced cellular immune responses, resulting in better protection against non-encapsulated *Y*. *pestis*. Our results present a new strategy for improving the protective effect of live-attenuated *Y*. *pestis* vaccines. In conclusion, we propose that EV76Δ*yp2* is a promising live-attenuated plague vaccine candidate that offers improved safety and efficacy when compared to EV76.

## Materials and methods

### Bacterial strains and growth conditions

Bacterial strains used in this study were shown in S1 Table. *Y*. *pestis* strains, 201 and EV76 vaccine, were routinely grown in Luria Bertani (LB) broth medium at 26°C. Bacteria were cultured at 26°C until an optical density (OD) of approximately 0.6 at 620 nm, followed by an incubation at 37°C for additional 3 hours prior to Western blotting analysis or preparation of bacterial lysates (201-WCL or 201Δ*caf1*-WCL). LB plates containing 7% sucrose and *Yersinia* selective agar (BD, Voigt Global Distribution Inc., Lawrence, KS) were used for *sacB* gene-based counter-selection in allelic exchange experiments for mutant constructions. *Escherichia coli* DH5α and *E*. *coli* S17-1 λpir were usually cultivated at 37°C in LB or on LB agar plates as plasmid donor strains. Kanamycin at 50 g/ml, ampicillin at 100 g/ml, and chloramphenicol at 25 g/ml were added as antibiotic supplements to the culture medium when necessary.

### Construction of the mutant strains of EV76

*Y*. *pestis* mutants were constructed using the λRed-based recombinant system as previously reported [66]. Briefly stated, kanamycin resistant cassettes from the plasmid pKD4 were amplified using primer sets *yp1*-P1/*yp1*-P1 and *yp2*-P1/*yp2*-P2 (S2 Table), respectively, to create EV76Δ*yp1* and EV76Δ*yp2*. In order to replace the *yp1* and *yp2* with the kanamycin resistance cassette, the PCR products were purified and electroporated into the competent bacterial cells. The cassette was later eliminated by introducing pCP20 [66], generating EV76Δ*yp1* and EV76Δ*yp2*. The primer sets Δ*yp1*-F/Δ*yp1*-R and Δ*yp2*-F/Δ*yp2*-R were utilized to further verify the strains.

A double mutant of EV76 was constructed using a suicide vector as previously reported [67]. Using the primer sets pre-*yp1*-F/ pre-*yp1*-R and post-*yp1*-F/ post-*yp1*-R, the homologous arm DNA segments from *Y*. *pestis* were amplified and cloned into *Sph*I and *Sac*I sites of pDS132, generating pDS132-*yp1*. The suicide plasmid pDS132-*yp1* was then introduced to EV76Δ*yp2* from *E*. *coli* S17-1 λpir by conjugation. On LB agar plates containing chloramphenicol, clones with the correct insert were identified, and the suicide vector was then removed through the second recombination by *sacB* counter-selection. PCR was used to confirm the clones with correct mutation using the primers Δ*yp1*-F/Δ*yp1*-R.

### Animal experiments

All the animal experiments were reviewed and approved by The Institute of Animal Care and Use Committee of the Academy of Military Medical Sciences (IACUC-DWZX-2020-071). 6- to 8-week-old female BALB/c mice were purchased from Vital River Laboratories (Beijing, China).

#### 1. Survival analysis of mice inoculated with different mutant strains of EV76

Groups of mice (n = 10 per group for individual strain) were *s*.*c*. inoculated with 1×10^7^ CFU of EV76 or different mutant strains. The mice were monitored for 14 days and their weight was measured daily after infection.

#### 2. Analysis of bacterial virulence to mice under iron overload conditions

Groups of mice (n = 10 per group for individual strain) were *s*.*c*. inoculated with 1×10^7^ CFU of EV76 and different mutant strains. Each group of mice was randomly divided into two subgroups. A daily intraperitoneal (*i*.*p*.) injection of 100 μl 0.1% FeCl_2_ (100μg) buffer or sterile deionized water was administered to each of the subgroups [32]. The survival rate of the mice was evaluated after 14 days of observation.

#### 3. Determination of bacterial loads in organs

Groups of mice (n = 30 per group for individual strain) were inoculated *s*.*c*. with 1×10^7^ CFU of EV76 and different mutant strains. 10 mice from each group were humanly euthanized at 1, 3, and 6 dpi. Inguinal lymph nodes and spleen were then aseptically extracted for further analysis. Using the MagNA Lyser (Roche, Germany), tissue samples were homogenized in 800 μl PBS. The homogenate was serially diluted in PBS and plated on *Y*. *pestis* Hettinger’s agar media plates (Hope Bio-Technology Co., Qingdao, China) to count bacterial numbers in different organs of mice administrated with different strains post 3 days’ incubation at 26°C [43].

#### 4. Animal immunization

The overnight cultures of EV76 and different mutant strains were inoculated into fresh LB broth and allowed to grow at 26°C for 12 h. Bacterial cultures were then re-inoculated into fresh LB broth and cultivated at 26°C with shaking at 200 rpm until reaching an OD of approximately 1.0 at 620 nm. Bacterial cells were harvested, washed, and diluted in PBS to OD_620_≈1.0. The bilateral groins of mice were inoculated *s*.*c*. with 100 μl with bacterial suspensions. The actual bacterial number was calculated by plating serial dilutions on *Y*. *pestis* Hettinger’s Agar plates.

#### 5. Evaluation of protection efficacy

For the *s*.*c*. challenge with *Y*. *pestis* 201, 201Δ*caf1*, or 201-*lux*. the bacteria strains were inoculated into LB and cultivated at 26°C until reaching an OD_620_ of approximately 1.0. For the *i*.*n*. challenge with the aforementioned strains, the bacteria were inoculated into LB and cultivated at 26°C until an OD_620_ of approximately 0.6, followed by an additional 2 h incubation at 37°C. Bacterial cells were harvested by centrifugation and the pellets were resuspended in sterile PBS. Groups of immunized mice were *s*.*c*. challenged. with 100μl suspensions of *Y*. *pestis* 201, 201Δ*caf1*, or 201-*lux* at appropriate concentrations, respectively. For *i*.*n*. challenge, immunized mice were anesthetized with iso pentobarbital (1.4mg/each) and then infected via nostril with 10 μl suspensions of *Y*. *pestis* 201, 201Δ*caf1*, or 201-*lux* suspension at appropriate concentrations.

*5*.*1 Evaluation of short-term protection efficacy*. Groups of mice (n = 10 per group for individual strain) were *s*.*c*. immunized with 1×10^7^ CFU of EV76 and different mutant strains, whereas mice injected with sterile PBS served as the control group. On day 21 post-immunization, immunized mice were subjected to *i*.*n*. challenged with *Y*. *pestis* 201 as described above. To evaluate the effectiveness of a single dose immunization in providing protection against pneumonic plague, mortality and morbidity of infected mice were monitored daily for the following 14 days.

Mice in various groups were immunized *s*.*c*. twice at a 21-day interval with 5×10^6^ CFU/dose of EV76 and different mutant strains (n = 20 per group for each strain, n = 10 in the control group). Immunized mice were subjected to *s*.*c*. or *i*.*n*. challenged with various dosages of *Y*. *pestis* 201 as above on day 42 following their initial immunization. Only the low dose of *Y*. *pestis* 201 was administered by *s*.*c*. or *i*.*n*. to the unvaccinated control group. The mortality and morbidity of infected mice were recorded daily for the following 14 days to evaluate the short-term protective effects of different vaccine candidates on mice.

In order to assess the potential effectiveness of vaccine candidates against *Y*. *pestis* 201Δ*caf1* in mice, the two-dose immunization strategy was also adopted. Immunized mice were subjected to *i*.*n*. challenged with 7×10^4^ CFU of *Y*. *pestis* 201. Mortality and morbidity of infected mice were monitored daily for the following 14 days.

*5*.*2*. *Evaluation of long-term protection efficacy*. Groups of mice (n = 15 per group for each strain or the control group) were *s*.*c*. vaccinated twice with 5×10^6^ CFU/dose of EV76 and different mutant strains at a 21-day interval. On day 120 post-initial immunization,10 mice from each immunized group were subjected to *s*.*c*. or *i*.*n*. challenge with *Y*. *pestis* 201-*lux*. Mortality and morbidity of the infected mice were consciously observed daily for the next 14 days. On days 123 and 130 post-immunization (day 3 and 12 post-infection), the animals were imaged by using IVIS (Spectrum, PerkinElmer, USA) to examine the dissemination and progress of infection.

### Histopathology

Group of mice (n = 10 per group for individual strain) were *s*.*c*. infected with 1×10^7^ CFU of EV76 and different mutants. The inguinal lymph nodes, lungs, spleens, and livers of five mice from each group were collected at 3 and 6 dpi. The organ tissues were fixed in 4% paraformaldehyde and then sliced, mounted on slides, and stained with hematoxylin-eosin (HE). Pathological alterations in the tissue slices were observed using light microscopy. A pathologist with specialized training performed blinded evaluation and assigned pathology scores to tissue sections according to the "International Harmonization of Nomenclature and Diagnostic Criteria for Lesions in Rats and Mice (INHAND)”: 0, no pathological lesions; 1, minimal; 2, mild; 3, moderate; 4, severe. [43]. The abnormal scores in the inguinal lymph nodes comprised congestion, necroptosis, inflammatory cell infiltration, and hyperplasia. Scores for spleens included extramedullary hematopoiesis, inflammatory cell infiltration, expansion of germinal centers, an increase of multinucleated giant cells, and congestion. The liver scores comprised hyperplasia, extramedullary hematopoiesis, inflammation cell infiltration, congestion, and degeneration of hepatocytes. Lastly, the lung scores were based on the degree of pulmonary alveolar wall thickening, inflammatory cell infiltration, congestion, and hemorrhage.

### Detection of antibody responses by ELISA and immunoblotting

On day 41 post first immunization, sera were collected from 10 immunized mice per group (2 doses, 5×10^6^ CFU/dose, 21 days apart). The levels of IgG that recognize F1, LcrV, or antigens in sonicated *Y*. *pestis* 201Δ*caf1* were evaluated by ELISA as previously described [43]. The sonicate of the *Y*. *pestis* 201Δ*caf1* strain was obtained by sonication of bacteria cells grown at 37°C in LB broth. Briefly, 96 well enzyme-linked plates (Costar, Corning, NY) were individually coated overnight with 2 μg/ml of rF1, 1μg/ml of rLcrV, or 10μg/ml of sonicated *Y*. *pestis* 201Δ*caf1*. After that, 2% bovine serum albumin (BSA) was used to block the 96 well plates.

Serial 2-fold dilutions of serum samples were performed. The diluted serum was next individually applied to wells, and the wells were then incubated at 37°C for 30 min with the appropriate antigen. The plates were then washed 5 times with PBS containing 0.1% Tween-20 (PBST), 100 μl of HRP-conjugated sheep anti-mouse IgG, or anti-mouse IgG1, IgG2a, IgG2b (Thermofisher, Vienna, Austria) were then added, followed by incubation at 37°C for 20 min. After an additional 5 washes, antibody titers were detected by a TMB substrate kit and analyzed with an iMark plate reader (Bia-Rad) at 450/630 nm. Background values were obtained from samples collected from the untreated mice. The titers of specific antibodies were calculated as the reciprocal of the OD_620_ value of the lowest sample dilution that produced a signal 2.1 times higher than that of background.

For immunoblotting analysis, the bacterial cell pellets were weighed to ensure equal amounts of *Y*. *pestis* 201 and 201Δ*caf1* bacterial cells were used to prepare the WCLs. Identical volumes of 201-WCL and 201Δcaf1-WCL were loaded onto 12% sodium dodecyl sulfate–polyacrylamide gel electrophoresis (SDS-PAGE). Separated proteins were transferred onto polyvinylidene difluoride (PVDF) membranes (Cytiva, Germany). Before being used as a primary antibody, serum samples collected from each group 41 days post-immunization (comprising equal volumes of serum from 10 mice) were diluted to a 1:100 ratio with TBST. The membrane was then incubated with the IRDye 800CW-conjugated goat-anti mouse secondary antibody and the immunoblotting results were imaged by an Odyssey SA imaging system (LI-COR Biosciences). as described previously [68].

### Detection of Antigen-specific T-cell responses

On day 41 after first immunization, 5 mice per group had their spleens as single cells, which were plated into 24-wells plates (Corning, Corning, NY) with RPMI 1640 medium (Gibco, Grand Island, NY) containing 10% FCS at a concentration of 5×10^6^ cells per well. The T cells in each well were then stimulated with 16 μg of sonicated *Y*. *pestis* 201-WCL or 201Δ*caf1*-WCL at 37°C in a 5% CO_2_ incubator. After 48 hours, culture supernatant from each well was collected to detect cytokines using Th1/Th2/Th9/Th17/Th22/Treg Cytokine 17-Plex Mouse Panel kits (Thermofisher, Vienna, Austria).

### Statistical analyses

All statistical analyses were conducted using GraphPad Prism version 8.0.1. Differences in bacterial load, antibody levels, and cytokine levels among various immunization groups were evaluated using one-way or two-way ANOVA, with subsequent *Tukey’s* or *Dunnett’s post hoc* tests. The Kruskal-Walli’s test, followed by Dunn’s post hoc test, was utilized to analyze pathological impairment scores. The animal survival rate was analyzed using Kaplan–Meier survival estimates. *P* < 0.05 was considered significantly different for all statistical analyses.

## Supporting information

S1 Table Strains and plasmids used in this study. (DOCX)

S2 Table Primers used in this study. (DOCX)

S3 Table The LD_50_ of *Y*. *pestis* 201, 201Δ*caf1* or 201-*lux* in BALB/c mice exposed via whole body aerosol or subcutaneous challenge. (DOCX)

S1 Fig Construction of mutant strains and expression of their major protective antigens. **(A)** *yp2* gene of the mutant strains was identified through PCR using outer primers (including *yp*2-F and *yp2*-R). **(B)** *yp1* gene of the mutant strains was identified through PCR using outer primers (including yp1-F and yp1-R). The amplification length of gene *yp1* in EV76Δ*yp1* and EV76Δ*yp1&yp2* is different because different gene editing techniques were used. (**C**) Expression of F1 antigen. (**D**) Expression of LcrV antigen.
(TIF)

S2 Fig Survival curves of mice *s*.*c* challenged with *Y*. *pestis* 201 mutant strains. (TIF)

S3 Fig Cellular immune responses to 201Δ*caf1* in mice administrated *s*.*c*. with EV76-derived candidate vaccines. Splenocytes were harvested and stimulated with 201Δ*caf1*-WCL and the supernatants were evaluated for cytokine secretion by Luminex assay (n = 5 for each group). One-way ANOVA with Tukey *post hoc* was used to determine the significance of differences. *P<0.05, **P<0.01, ***P<0.001, ****P<0.0001
(TIF)

S1 Data Raw data for figures. (XLSX)
