# Supplementary material for: A novel sORF gene mutant strain of Yersinia pestis vaccine EV76 offers enhanced safety and improved protection against plague
Source: PLoS Pathog. 2024 Mar 28;20(3):e1012129. doi: 10.1371/journal.ppat.1012129 (PMC11020802; doi:10.1371/journal.ppat.1012129)
Supplement: S1 Table — (DOCX) [file ppat.1012129.s001.docx]

S1 Table. Strains and plasmids used in this study

| **Strains or plasmids** | **Descriptions** | **Sources** |
| --- | --- | --- |
| **Strains** |  |  |
| *E. coli* S17-1 λpir | contains the pir gene (λpir), have chromosomally integrated conjugal transfer functions. | Laboratory collection |
| *E. coli* DH5α | F-φ80 *lacZ*ΔM15 Δ(*lacZYA*-arg F) U169 *endA1 recA1 hsdR17* (*rk*^-^, *mk*^+^) *supE44* λ- *thi-1* *gyrA96 relA1 pho* | Purchased from the company |
| *Y. pestis* EV76 | *pgm^-^* | Laboratory collection |
| *Y. pestis* EV76Δ*yp1* | *pgm^-^, yp1* gene was replaced by 85nt DNA scar | This study |
| *Y. pestis* EV76Δ*yp2* | *pgm^-^, yp2* gene was peplaced by 85nt DNA scar | This study |
| *Y. pestis* EV76Δ*yp1&yp2* | *pgm^-^,* Δ*yp1, yp2* gene was peplaced by 85nt DNA scar | This study |
| *Y. pestis* 201 | Wild-type *Y. pestis* strain, avirulent to humans, highly virulent to mice | Laboratory collection |
| *Y. pestis* 201Δ*yp2* | *yp2* gene was replaced by 85nt DNA scar | Laboratory collection |
| *Y. pestis* 201Δ*yp1&yp2* | Δ*yp1, yp2* gene was replaced by 85nt DNA scar | This study |
| *Y. pestis* 201Δ*caf1* | unable to express F1 antigen | Laboratory collection |
| *Y. pestis* 201-*lux* | *luxCDABE* was inserted into the chromosomes of 201 | ^[1]^ |
| **Plasmids** |  |  |
| pKD46 | Temperature-sensitive plasmid expressing λRed recombinase under the control of arabinose; Ap^R^ | Laboratory collection |
| pKD4 | Template plasmid carrying antibiotic resistance genes that are flanked by FRT sites. | Laboratory collection |
| pCP20 | an Ap^R^ and Cm^R^ plasmid that shows temperature-sensitive replication and thermal induction of FLP synthesis | Laboratory collection |
| pDS132 | Suicide vector, Cm^R^, 6K ori, mobRP4, *sacB* | Laboratory collection |
| pDS132-*yp1* | The Δ*yp1* fragment was cloned into pDS132 | This study |

1. Zhou J, Bi Y, Xu X, Qiu Y, Wang Q, Feng N, et al. Bioluminescent tracking of colonization and clearance dynamics of plasmid-deficient *Yersinia pestis* strains in a mouse model of septicemic plague. Microbes Infect. 2014;16(3):214-24. Epub 2013/12/18. doi: 10.1016/j.micinf.2013.11.013. PubMed PMID: 24333143.
