# Supplementary material for: A novel sORF gene mutant strain of Yersinia pestis vaccine EV76 offers enhanced safety and improved protection against plague
Source: PLoS Pathog. 2024 Mar 28;20(3):e1012129. doi: 10.1371/journal.ppat.1012129 (PMC11020802; doi:10.1371/journal.ppat.1012129)
Supplement: S2 Table — (DOCX) [file ppat.1012129.s002.docx]

S2 Table. Primers used in this study

| Name | Sequence（5’-3’） |  |
| --- | --- | --- |
| *yp2*-P1 | ***TCAGAAAATAGACAGTGTTGACACGCGCTCACACTATACCTATCATAATGA***TGTGTAGGCTGGAGCTGCTTC |  |
| *yp2*-P2 | ***CTGGTTTTTGATGCGACCACACTGGTCGACCGCTTGCCTGATTTGCGTGGCATGGTGCTCCGGA***CATATGAATATCCTCCTTA |  |
| Δ*yp2*-F | CTATAGCCGTTTTCTGCGGC |  |
| Δ*yp2*-R | CCTTTGGAACAAATGGCGGC |  |
| *yp1*-P1 | ***CATGCCATGCATGAGTCCAAAACCCCGCTAATATAGTTAG***TGTGTAGGCTGGAGCTGCTTC |  |
| *yp1*-P2 | ***TTAAATGCTTAAATTAAGAGTGATGACACCCAAACCCAAG***CATATGAATATCCTCCTTA |  |
| Δ*yp1*-F | ACAAGCATTTAAAGACCAAAGCC |  |
| Δ*yp1*-R | ACTGGACGTGACCTAGAAGT |  |
| pCP20-F | TTGATGCGCTGGCAGTGTTC |  |
| pCP20-R | GAGCTTTGTTGTAGGTGGACC | |
| pre-*yp1*-F | ***AAGCTTCTTCTAGAGGTACC*gcatgc**TGTTCATACTCAATATCCGC  (*Sph*Ⅰ) | |
| pre- *yp1*-R | ***GATGACACCCAAACCCAAG***CTAACTATATTAGCGGGGTT |  |
| post- *yp1*-F | ***ATATAGTTAG***CTTGGGTTTGGGTGTCATCA |  |
| post- *yp1*-R | ***ATTTGTGGAATTCCCGGGA*gagctc**TATTGGGCAGGCACTGGTCT(*Sac*Ⅰ) |  |

Bold, italicized, underlined capital letters indicate homologous arm sequences
