# Supplementary material for: A novel sORF gene mutant strain of Yersinia pestis vaccine EV76 offers enhanced safety and improved protection against plague
Source: PLoS Pathog. 2024 Mar 28;20(3):e1012129. doi: 10.1371/journal.ppat.1012129 (PMC11020802; doi:10.1371/journal.ppat.1012129)
Supplement: S3 Table — (DOCX) [file ppat.1012129.s003.docx]

S3 Table. The LD_50_ of *Y. pestis* 201, 201Δ*caf1* or 201-*lux* in BALB/c mice exposed via intranasal or subcutaneous challenge

| **Bacterial strain** | **Route of exposure** | **LD_50_ (CFU)^a b^** |
| --- | --- | --- |
| *Y. pestis* 201 | Intranasal | 438 |
| *Y. pestis* 201Δ*caf1* | Subcutaneous | 2.48 |
| *Y. pestis* 201Δ*caf1* | Intranasal | 338 |
| *Y. pestis* 201-*lux* | Subcutaneous | 2.37 |
| *Y. pestis* 201-*lux* | Intranasal | 859 |
| ^a^ colony-forming unit |  |  |
| ^b^ The confidence interval was unbounded. | | |
