# Supplementary figures and images for: A novel sORF gene mutant strain of Yersinia pestis vaccine EV76 offers enhanced safety and improved protection against plague
Source: PLoS Pathog. 2024 Mar 28;20(3):e1012129. doi: 10.1371/journal.ppat.1012129 (PMC11020802; doi:10.1371/journal.ppat.1012129)

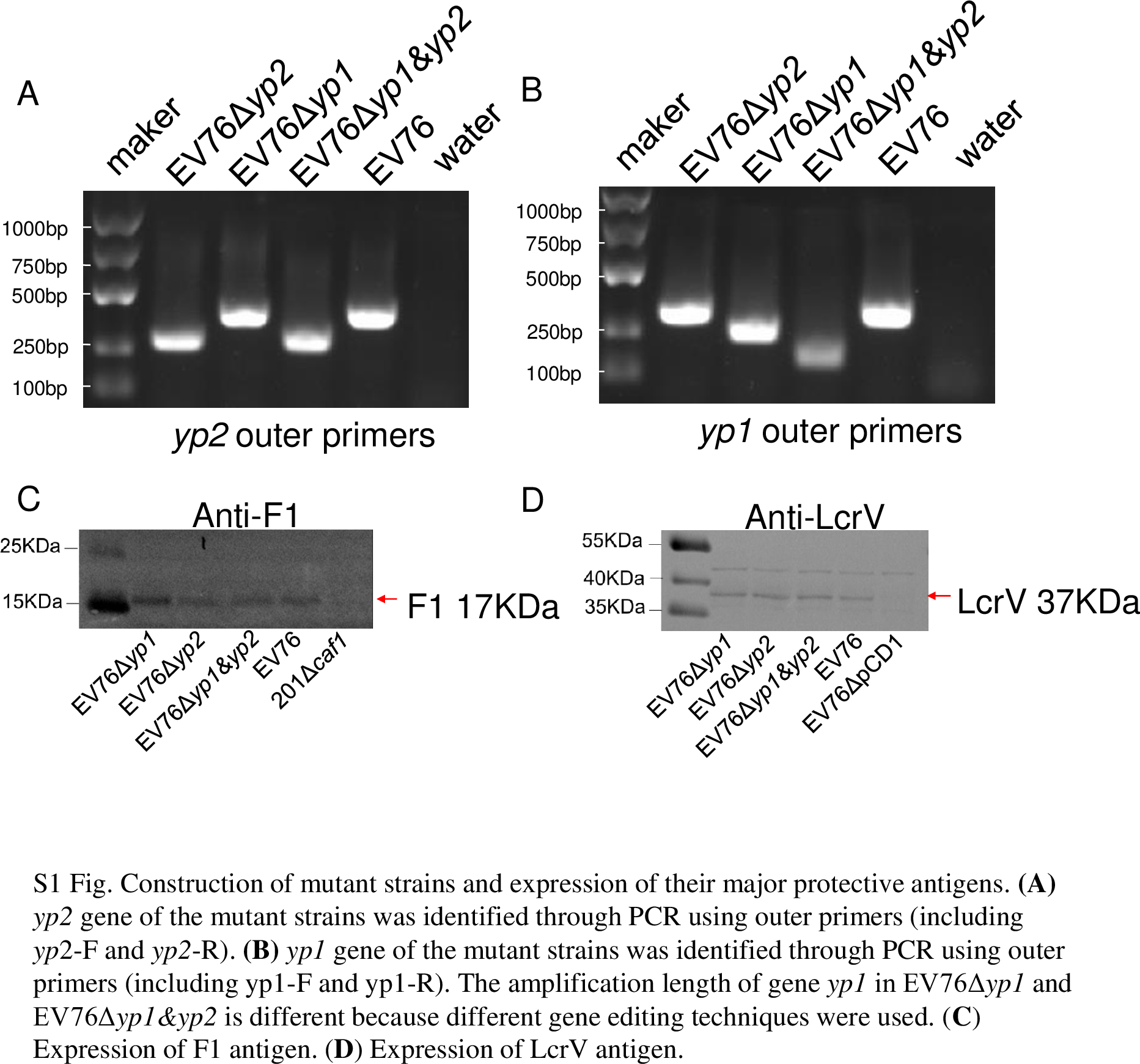

Supplement: S1 Fig — (A) yp2 gene of the mutant strains was identified through PCR using outer primers (including yp2-F and yp2-R). (B) yp1 gene of the mutant strains was identified through PCR using outer primers (including yp1-F and yp1-R). The amplification length of gene yp1 in EV76Δyp1 and EV76Δyp1&yp2 is different because different gene editing techniques were used. (C) Expression of F1 antigen. (D) Expression of LcrV antigen. (TIF) [file ppat.1012129.s004.tif]

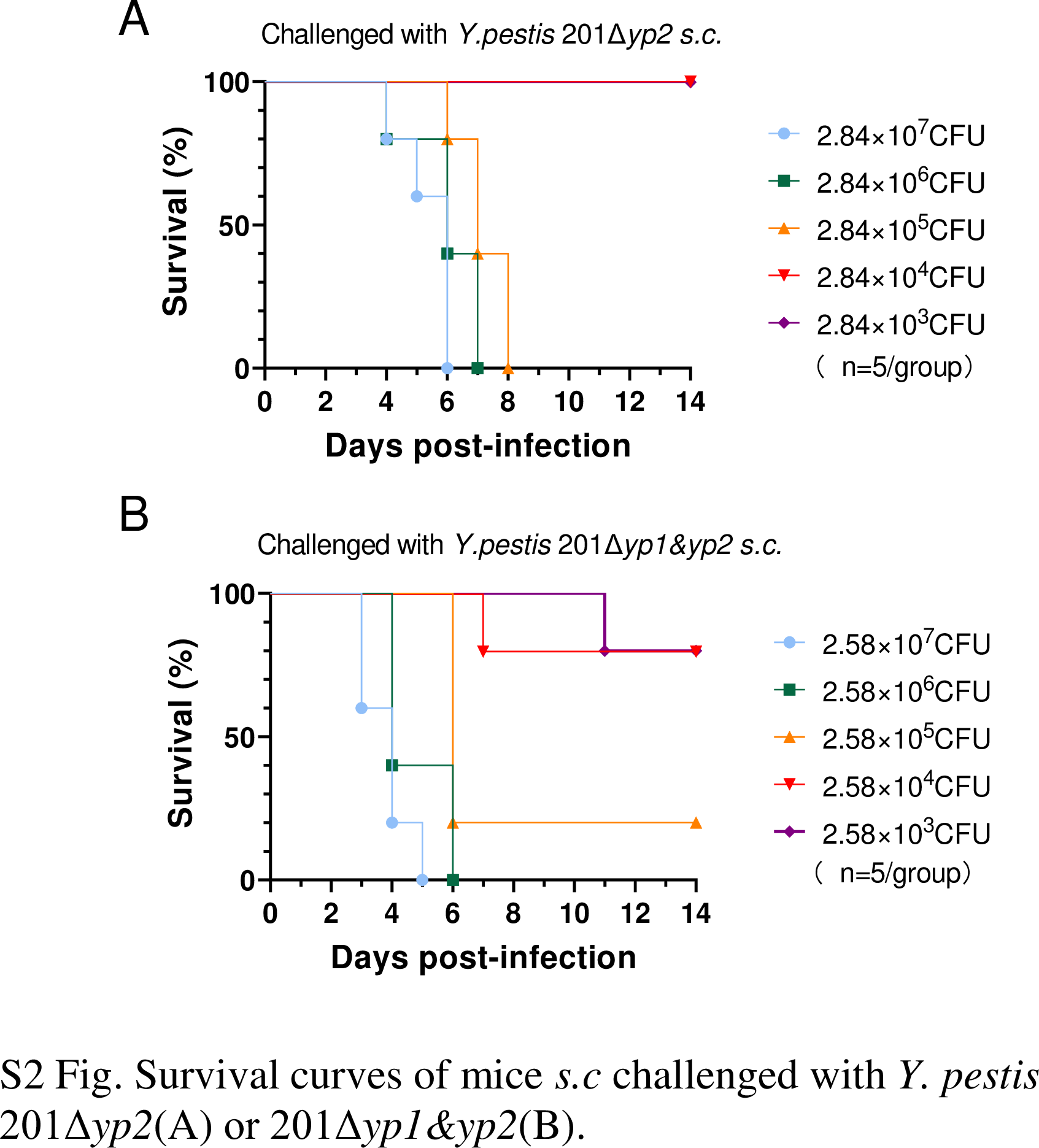

Supplement: S2 Fig — (TIF) [file ppat.1012129.s005.tif]

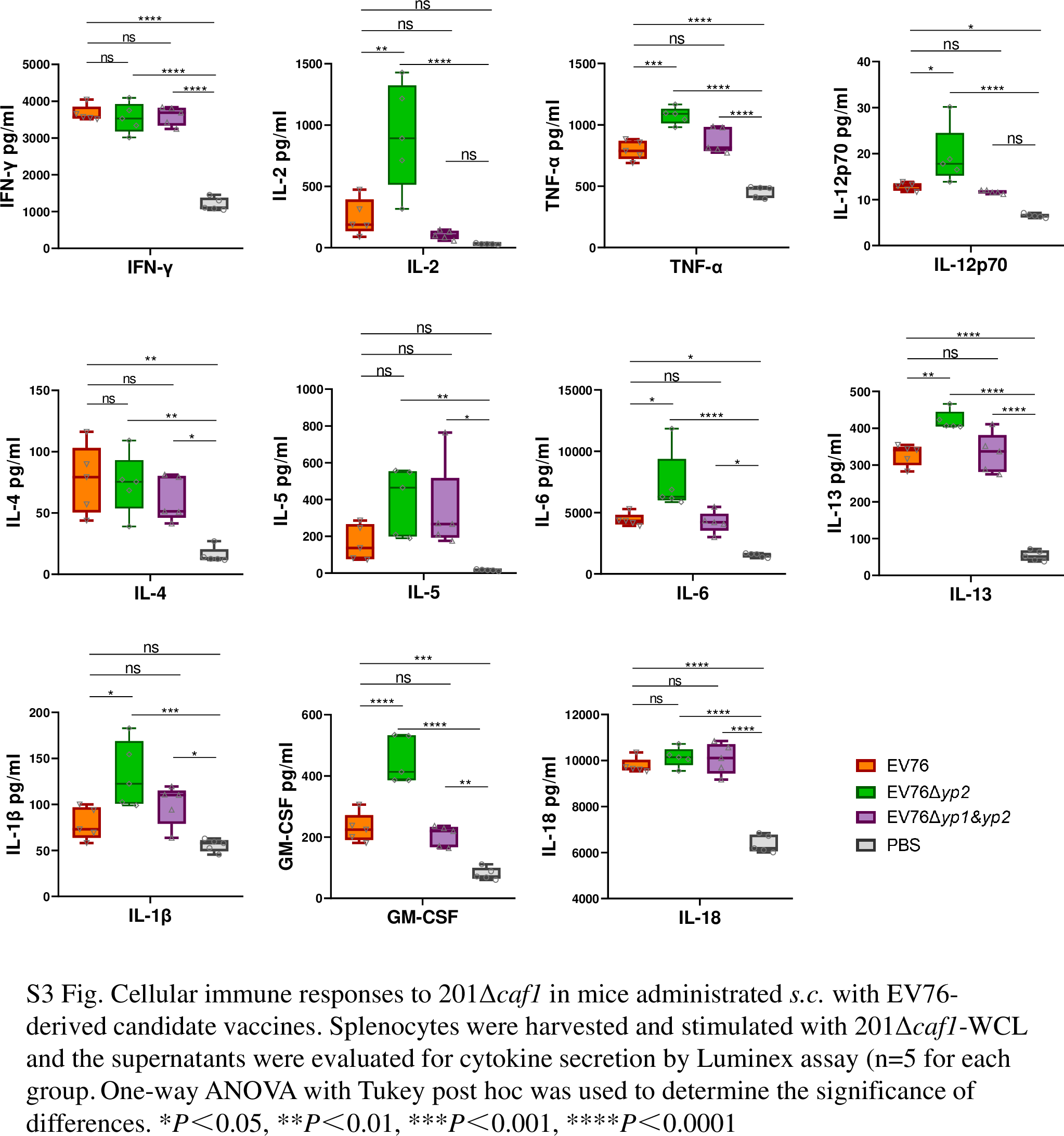

Supplement: S3 Fig — Splenocytes were harvested and stimulated with 201Δcaf1-WCL and the supernatants were evaluated for cytokine secretion by Luminex assay (n = 5 for each group). One-way ANOVA with Tukey post hoc was used to determine the significance of differences. *P<0.05, **P<0.01, ***P<0.001, ****P<0.0001 (TIF) [file ppat.1012129.s006.tif]
